# High-Resolution Maps of Material Stocks in Buildings and Infrastructures in Austria and Germany

**DOI:** 10.1021/acs.est.0c05642

**Published:** 2021-02-18

**Authors:** Helmut Haberl, Dominik Wiedenhofer, Franz Schug, David Frantz, Doris Virág, Christoph Plutzar, Karin Gruhler, Jakob Lederer, Georg Schiller, Tomer Fishman, Maud Lanau, Andreas Gattringer, Thomas Kemper, Gang Liu, Hiroki Tanikawa, Sebastian van der Linden, Patrick Hostert

**Affiliations:** † Institute of Social Ecology, University of Natural Resources and Life Sciences, Vienna, Schottenfeldgasse 29, 1070 Vienna, Austria; ‡ Geography Department, Humboldt Universität zu Berlin, Unter den Linden 6, 10099 Berlin, Germany; § Integrative Research Institute on Transformations of Human-Environment Systems, Humboldt Universität zu Berlin, Unter den Linden 6, 10099 Berlin, Germany; ∥ Department of Botany and Biodiversity Research, University of Vienna, Rennweg 14, 1030 Wien, Austria; ⊥ Leibniz Institute of Ecological Urban and Regional Development, Weberplatz 1, D-01217 Dresden, Germany; # Institute for Water Quality and Resource Management, TU Wien, Karlsplatz 13/226.2, A-1040 Wien, Austria; ∇ Institute of Chemical, Environmental and Bioscience Engineering, TU Wien, Getreidemarkt 9/166, A-1060 Wien, Austria; ○ School of Sustainability, Interdisciplinary Center (IDC) Herzliya, Hauniversita 8, 4610101 Herzliya, Israel; ◆ SDU Life Cycle Engineering, Department of Green Technology, University of Southern Denmark, 5230 Odense, Denmark; ¶ Department of Civil and Structural Engineering, University of Sheffield, Sir Frederick Mappin Building, Mappin Street, S1 3JD Sheffield, U.K.; ⋈ European Commission, Joint Research Centre, Via E. Fermi 2749, 21027 Ispra, VA, Italy; ⧓ Department of Environmental Engineering and Architecture in the Graduate School of Environmental Studies, Nagoya University, 464-8601 Nagoya, Japan; ⧖ Institut für Geographie und Geologie, Universität Greifswald, Friedrich-Ludwig-Jahn-Str. 16, D-17489 Greifswald, Germany

## Abstract

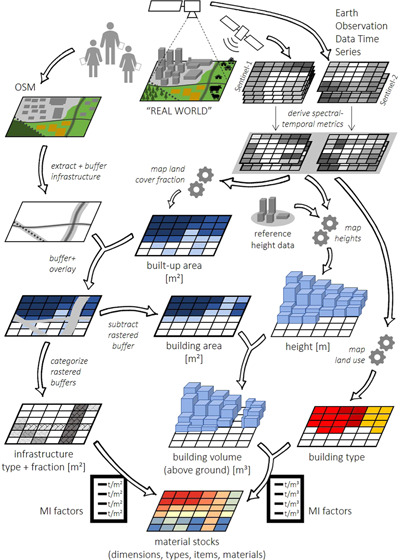

The dynamics of societal material stocks such as buildings and infrastructures and their spatial patterns drive surging resource use and emissions. Two main types of data are currently used to map stocks, night-time lights (NTL) from Earth-observing (EO) satellites and cadastral information. We present an alternative approach for broad-scale material stock mapping based on freely available high-resolution EO imagery and OpenStreetMap data. Maps of built-up surface area, building height, and building types were derived from optical Sentinel-2 and radar Sentinel-1 satellite data to map patterns of material stocks for Austria and Germany. Using material intensity factors, we calculated the mass of different types of buildings and infrastructures, distinguishing eight types of materials, at 10 m spatial resolution. The total mass of buildings and infrastructures in 2018 amounted to ∼5 Gt in Austria and ∼38 Gt in Germany (AT: ∼540 t/cap, DE: ∼450 t/cap). Cross-checks with independent data sources at various scales suggested that the method may yield more complete results than other data sources but could not rule out possible overestimations. The method yields thematic differentiations not possible with NTL, avoids the use of costly cadastral data, and is suitable for mapping larger areas and tracing trends over time.

## 1 Introduction

Transformations toward sustainable low-carbon societies and the Sustainable Development Goals (SDGs) require far-reaching changes in societies’ use of biophysical resources such as energy, materials, or land.^1−5^ Researchers study patterns of resource use (“social metabolism”) using methods of Material and Energy Flow Analysis.^6^ These analyses focus on socioeconomic flows of energy, materials, or substances, underpinning research on eco-efficiency^7^ and long-term socioecological transitions.^8^ Societal material and energy flows are relevant for sustainability due to a plethora of systemically interrelated concerns such as the depletion of nonrenewable resources, ecological degradation from resource extraction, and wastes or emissions resulting in climate change and other detriments.^6,9^

Sociometabolic flows are required to build up, operate, and maintain societies’ biophysical structures, such as buildings, infrastructures, or machinery. These structures are usually denoted as “artifacts”,^10^ “manufactured capital”,^11,12^ “technomass”,^13,14^ “in-use stocks of materials”,^15,16^ or “material stocks”;^17,18^ we here use the latter term. Over the last decade, the quantification of material stocks has received increasing attention.^15,19−21^

Material stocks have important environmental impacts due to their land demand^22−24^ and GHG emissions,^15,25,26^ but they also play a pivotal role in transforming resource flows into services such as shelter, nutrition, or mobility.^11^ Building up and maintaining stocks require large amounts of resources; currently, stock-building materials amount to almost 60% of all materials used by humanity.^12^ Buildings, infrastructures, and machinery shape social practices of production and consumption, thereby creating path dependencies for future resource use.^25^ They constitute the physical basis of the spatial organization of most socioeconomic activities, for example, as mobility networks, urbanization and settlement patterns, and various other infrastructures.^24,27^ Analyzing the inter-relations between material stocks, material and energy flows, and the services they provide to societies^17,28^ (the so-called “stock-flow-service nexus”^6,29^) provides a much richer picture than the traditional views of “decoupling” and eco-efficiency, which focus on resource use, waste, and emissions vis-a-vis economic activity (usually measured as the gross domestic product) as a basis of policies aiming to reduce resource use or emissions.^3,7,30^

Dynamic inflow-driven “top-down” approaches quantify material stocks by calculating inflows to stocks and subtracting outflows from stocks over long time periods.^21^ One such approach has been used to derive a centennial global time-series of material stocks,^12^ however, with almost no spatial differentiation (three world regions). While the country-level resolution is attainable with this approach,^31^ it is not suited for high-resolution mapping and hence misses a key characteristic of material stocks, i.e., how they distribute in space. Methods to map material stocks generally employ a stock-driven “bottom-up” approach: spatially explicit data are used to calculate physical dimensions of buildings and infrastructures, such as length, area, or volume. The mass of material stocks can be calculated with material intensity (MI) factors (kg/m, kg/m^2^, or kg/m^3^) that extrapolate the mass of buildings and infrastructures from their types and physical dimensions.^18,20,32−35^ So far, two main sources of spatially explicit data have been used for deriving material stock maps, both of which have advantages and disadvantages:

(1) Cadasters provide data at the level of individual buildings and infrastructure at very high resolution. They are usually derived from official digitalized city plans, building permits, and infrastructure mappings, sometimes even available as public three-dimensional (3D) city or neighborhood models,^36^ or obtained directly from lidar data.^32^ They allow for highly detailed calculations of material stocks both in terms of spatial resolution and distinction of different materials, given sufficient data.^20^ Cadasters can be used to generate very practicable information, for example, for spatial planning, urban mining, or waste management.^33,34,37−39^ Highly resolved lidar can provide data when cadaster data are unavailable, but these data are costly to obtain and often limited to relatively smaller areas such as city districts.^32^ Regardless of the source, however, this approach is very data-intensive, and cadastral data are often expensive, difficult to access, and sometimes not available. This confines most studies to specific areas or cities.
(2) Night-time light (NTL) data are derived from satellite imagery and are available globally.^40^ Because their radiance was found to correlate well with human activities, NTL data are widely used as a proxy for socioeconomic variables such as population, gross domestic product, or human development.^41−45^ NTL data are readily available for large areas, and data on the presence and intensity of night-time light are increasingly used to extrapolate material stocks.^46−48^ These methods generally rely on regression-based extrapolations from local or regional cadaster-based stock maps. However, deriving stock estimates from NTL data misses nonluminous structures and/or misinterpret luminous structures. Inaccuracies may further result from saturation effects of NTL, the underestimation of rural features, and even larger settlements in less-developed countries^49^ as well as a coarse spatial resolution, leading to an inability to identify specific building or infrastructure types with NTL.

We here demonstrate a new stock-driven mapping approach that combines the strengths of the aforementioned methods (high spatial resolution, large spatial coverage) and avoids their drawbacks (low resolution, small spatial coverage). This is possible using different sources of input data, namely advanced Earth observation (EO) products derived from optical Copernicus Sentinel-2 (S2) and radar Copernicus Sentinel-1 (S1) sensors as well as crowd-sourced data (Open Street Map, OSM). We combine these spatially explicit datasets with a comprehensive database of MI factors of five building types and 21 types of infrastructures, thereby distinguishing 13 types of materials. We aggregated these detailed data to three types of buildings (single-family houses, multifamily houses, industrial/commercial buildings), three types of infrastructures (high-level roads, all other roads, railways of various kinds), and seven material groups. Disaggregated data on building and infrastructure types as well as material categories are available in the Supporting Information (SI). The aim is to provide an approach that can be used to map material stocks for larger (national or continental) areas and that is potentially applicable to regions without cadasters or even at the global scale, while still providing a high level of spatial detail. We develop this novel approach for two countries, Austria [AT] and Germany [DE], where estimates of material stocks are partially existing for cross-comparison on national^50,51^ and local/regional^39,52,53^ levels, which facilitates derivation of MI factors and allows to compare results with previous estimates.

## 2 Methods and Data

**Figure 1:**
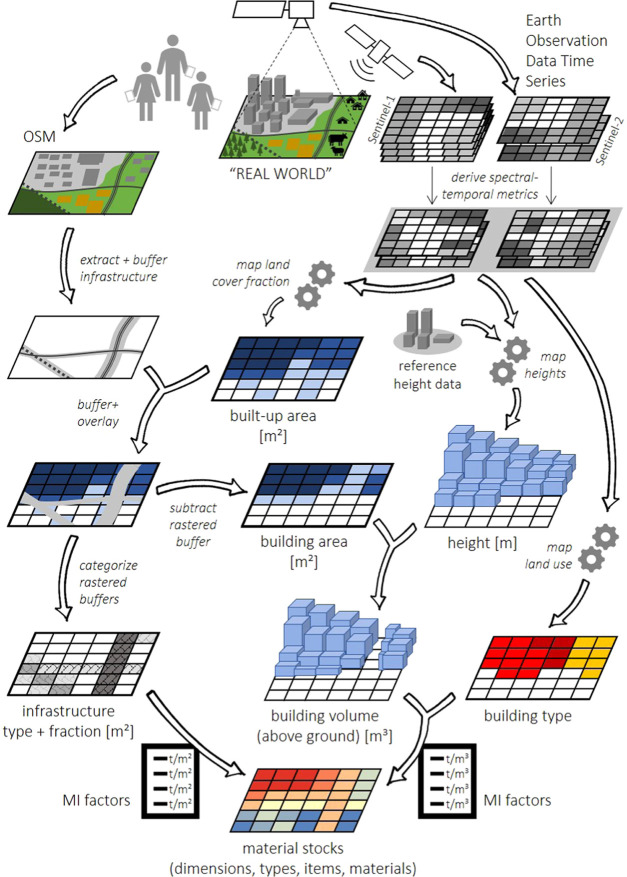
Data sources used and processing workflow employed in this study to generate material stock maps. Details on data resolution, calculations, and validation are provided in the text. Source: own illustration.

Our method for mapping material stocks combines three fundamentally different types and sources of data: (1) EO raster data that characterize built-up structures with regard to their density, vertical extent, and type derived from S1 and S2 satellite imagery with an initial spatial resolution of 10 m; (2) infrastructure data from crowd-sourced OSM vector data; and (3) tabular data on MI factors that give information on the amount [kg] of materials per unit area [m^2^] or volume [m^3^] of each specific type of infrastructure or building compiled from the literature. Figure 1 provides an overview of data sources and main processing steps that establish compatibility between these. Details on calculation procedures, resolution, and accuracy of data as well as their validation are discussed in Sections 2.1–2.3. More detailed documentation can be found in the SI.

### 2.1 Mapping Buildings and Infrastructures Using Geospatial Data

We used OSM data on roads and railways from Geofabrik.^54^ We extracted the line layers with the key “highway” and “railway” separately for AT and DE using the Osmium tool (https://osmcode.org/osmium-tool/). In order to estimate the width of “linear structures”—roads, railways, etc.—in OSM, we calculated averages using the “width” tag for 39 types of roads and 12 types of railways (including tram and metro lines). For infrastructure types that do not hold a width information in OSM, we relied on expert assessment (SI). On the basis of this information, we buffered all road and railway features in OSM to calculate their area coverage [m^2^]. Polygon and buffered line vector data on the infrastructure were rasterized and corrected for overlapping features that would result in an area share of infrastructure above 100%.

We quantified the subpixel share of the built-up area [m^2^] of buildings, infrastructure, and other human-made structures for cells of 10 × 10 m^2^ within the study area^55^ using a machine-learning-based regression approach.^56,57^ As input, we used optical S2^58^ time series for 2017/2018 from the European Commission’s Copernicus program, from which we derived metrics capturing the variability over time for various parts of the electromagnetic spectrum (e.g., visible light, near and short-wave infrared). We improved on Schug et al.^55^ by adding a proxy for green vegetation (Tasseled Cap Greenness^59^) as well as data from the microwave domain through the inclusion of S1 radar time series;^60^ see SI for reasoning. We validated the built-up area layer by manually labeling reference data at 160 sites, covering twenty-five 10 × 10 m^2^ cells each with a total of 36 000 samples.

To capture the vertical distribution of material stocks, we derived building height for each 10 × 10 m^2^ grid cell across the study area based on morphological metrics from S1 and S2 time series.^58,61^ A support vector regression was trained with building height derived from several highly accurate building height reference datasets across Germany.^61^ The reference data were obtained from official geodetic surveys (Table SI_1) and were generated by merging the building footprint cadaster with height measurements from laser scanning overflights. The building height represents the rooftop. A separate paper by some of the authors^61^ assessed the prediction accuracy using a 30% left-out sample and ensured the model application to unknown locations with a cross-validation approach by repeatedly comparing regression outputs against datasets not used for training the model (i.e., Potsdam, Hamburg, and the complete German federal states of North Rhine Westphalia and Thuringia). Similarly, the building height prediction was compared with the building height dataset of Vienna to ensure the regression’s applicability in Austria (SI).

We classified the types of buildings for each 10 × 10 m^2^ cell using classes for commercial and industrial buildings, single-family housing, multifamily housing, and lightweight buildings, a heterogeneous category of buildings mostly using a rigid frame construction, usually made of timber, e.g., car-ports, garages, sheds, or garden bungalows.^62^ The classification was based on morphological metrics from S1 and S2 that provide information on reflectance and backscatter characteristics of each pixel’s surroundings.^58^ We used morphological metrics as an input to a random forest classifier; 1604 training samples were collected across Germany using manual interpretation of ©Google Earth imagery. In a second step, high-rise buildings were separated from multifamily houses by employing a >30 m building height threshold.

To reduce commission errors (“false-positives”) of the built-up area estimation, we only considered pixels with a built-up area of more than 25 m^2^ per 100 m^2^. To retrieve building area, i.e., the share of the area of each 10 × 10 m^2^ grid cell covered by buildings, we subtracted the rasterized area of aboveground infrastructure from the built-up area. Subsequently, we derived a calibration factor, which was obtained from the linear relationship between provisional building area (already corrected for aboveground infrastructure) and cadastral reference data, showing that our Earth observation-based method systematically, but predictably, overestimates the building surface by a factor of 0.53 (see the SI). This calibration corrects for rather small flat infrastructures like private parking lots, which are not included in OSM, and thus, cannot be subtracted from the built-up surface along with the more prominent transportation infrastructure. It also corrects for building roof overhang. As the EO-based building type product is not capable of distinguishing single-family residential houses from attached garages, the building area of single-family houses was further reduced by 10%, and that area was added to the lightweight building category. The product of building area [m^2^] and building height [m] was computed to obtain aboveground building volume [m^3^] according to the volume definition shown in Figure SI_8. For the garage area in the lightweight class, we used a constant height of 2.7 m for the volume calculation (SI).

### 2.2 Material Intensities Database

The methods described in Section 2.1 yield data on the aboveground volume of buildings, simplified into a cube, and infrastructures (areal coverage); see Figure 1 and SI. These data are not consistent with some of the definitions used by researchers quantifying the mass of materials in buildings and infrastructures, and hence, we had to develop consistent MI factors. Building on previous work by some of the co-authors, we combined and recalculated several literature sources to derive a dataset of material intensity factors (MI) for 23 stock types and 13 materials (Table 1 and SI). For the three main building types in DE, this recalculation was based on the IOER database.^63^ We derived AT-specific MI factors for buildings from information on Vienna.^53^ For the six road types in both DE and AT, we used a combination of sources,^64−68^ which are listed below as weighted-average intensities for high-level roads, such as motorways and primary roads, and for all other roads including secondary, tertiary, service, and gravel roads. Railways,^64^ subways^52^ and trams^69^ could be derived directly from stock-type specific studies. For bridges, we used information from Vienna;^70^ the MI for tunnels is from Steger et al.^64^ Because only a small fraction of high-level roads is made from cement concrete, and no robust data were available, we neglected cement concrete in roads.

**Table 1:** Overview of Material Intensities Used for Estimating the Mass of Material Stocks^a^^,^^g^

	buildings						infrastructures					
	single-family residential buildings [kg/m^3^]		multifamily residential buildings [kg/m^3^]		commercial and industrial buildings [kg/m^3^]^f^		high-level roads (motorway, primary) [kg/m^2^]		all other roads [kg/m^2^]		railway [kg/m^2^]	
	AT^b^	DE^c^	AT^b^	DE^c^	AT^b^	DE^c^	AT^d^	DE^d^	AT^d^	DE^d^	AT^e^	DE^e^
metals	10.9	20.0	12.0	21.5	12.4	24.4	—	—	—	—	17.1	16.9
concrete	242	206	258	202	221	186	—	—	—	—	29.5	43.3
bricks	127.3	46.9	116.1	88.4	90.2	15.4	—	—	—	—	0.0	0.0
aggregate	29.7	33.1	28.6	23.0	24.6	113	1472	1501	956	956	270	285
other nonmetallic minerals	6.4	210	5.9	115	3.5	48.2	—	—	—	—	—	—
biomass materials	6.3	14.1	6.2	6.1	3.3	3.1	—	—	—	—	10.6	3.0
petrochemical-based materials	0.2	1.3	0.3	1.3	0.4	1.0	19.2	19.5	10.5	10.5	1.3	0.7
total	423	531	427	458	356	391	1491	1521	967	967	328	349

a Detailed documentation and disaggregated data for the four building types, six road types, three railway types as well as bridges and tunnels used in the mapping are in the SI.

b Ref (53).

c Ref (63).

d Refs (64−68).

e Refs (64, 71, 72).

f These MI factors do not comply with the same building definition; see the SI for details.

g Empty cells marked with “—” indicate that these materials are not present in the respective structure.

### 2.3 Validation and Uncertainty

Our novel approach combines new types of data from different research fields, and hence, validation and uncertainty assessment are not straightforward. We therefore opted for a step-by-step approach, i.e., validated results from intermediate steps against other available (also incomplete) data wherever possible. A comprehensive, systematic quantification of uncertainties was beyond the scope of this study. The intermediate remote-sensing mapping products described above were independently validated in the respective methodological publications,^55,61^ which we summarize here. The uncertainty of the land cover share estimate, measured as the root mean squared error (RMSE), was approximately 19% (for validation see the SI). The uncertainty of the height estimation (RMSE validated against six freely available local high-resolution 3D city models across the study area) was approximately 3-4 m when accounting for building height class frequencies; see the SI and ref (61). The building type classification was validated based on a 30% subsample of the collected training data; the overall accuracy for DE was found to be 81.40%;^62^ validation results for AT are in the SI. We compared the length of roads and railways reported in OSM against statistical data sources collated in ref (65) as well as the national statistical data.^73−75^ We compared results on the mass of material stocks by type and material against the available literature sources.^12,39,50−52,64,65,70,76−78^ Detailed documentation and discussion of the validations are available in the SI; findings are summarized in the Section 3.

## 3 Results

**Figure 2:**
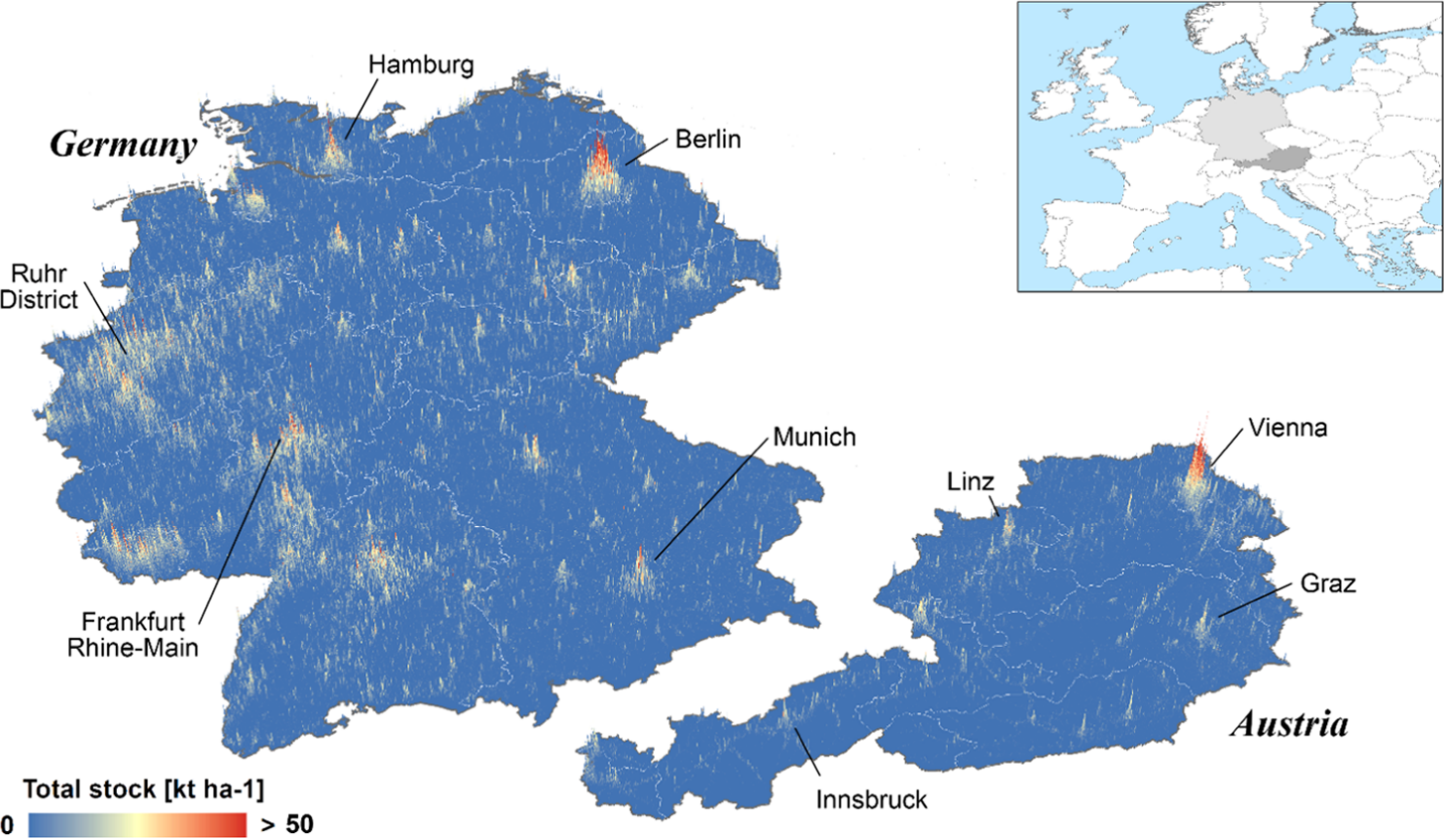
Three-dimensional maps of total material stocks in buildings and infrastructures in Germany and Austria (2018; 100 m resolution), measured as kt/ha (1 kt = 1000 metric tons; 1 ha = 10^4^ m^2^ = 0.01 km^2^).

Figure 2 shows the estimated distribution of total material stocks as a 3D-map at a spatial resolution of 100 m for our study area. This map only serves as a visual representation of our data, which are available for download at available for download for Austria at https://zenodo.org/record/4522892#.YCKJ1-hKhEY and for Germany at https://zenodo.org/record/4536990#.YCfPfGhKgmI. We observe a general pattern of stocks in areas where a high share of the built-up surface is to be expected: large urban agglomerations are visible as high peaks, sparsely settled areas such as the Alps in central Austria (AT) and southern Germany (DE), and rural regions in both countries have low levels of human-made material stocks, depicted blue in the maps. A two-dimensional (2D)-map with 100 m resolution is available in the SI (Figure SI_13). A web viewer that visualizes the total material stock map and per-state statistics is available at https://ows.geo.hu-berlin.de/webviewer/stocks.

**Figure 3:**
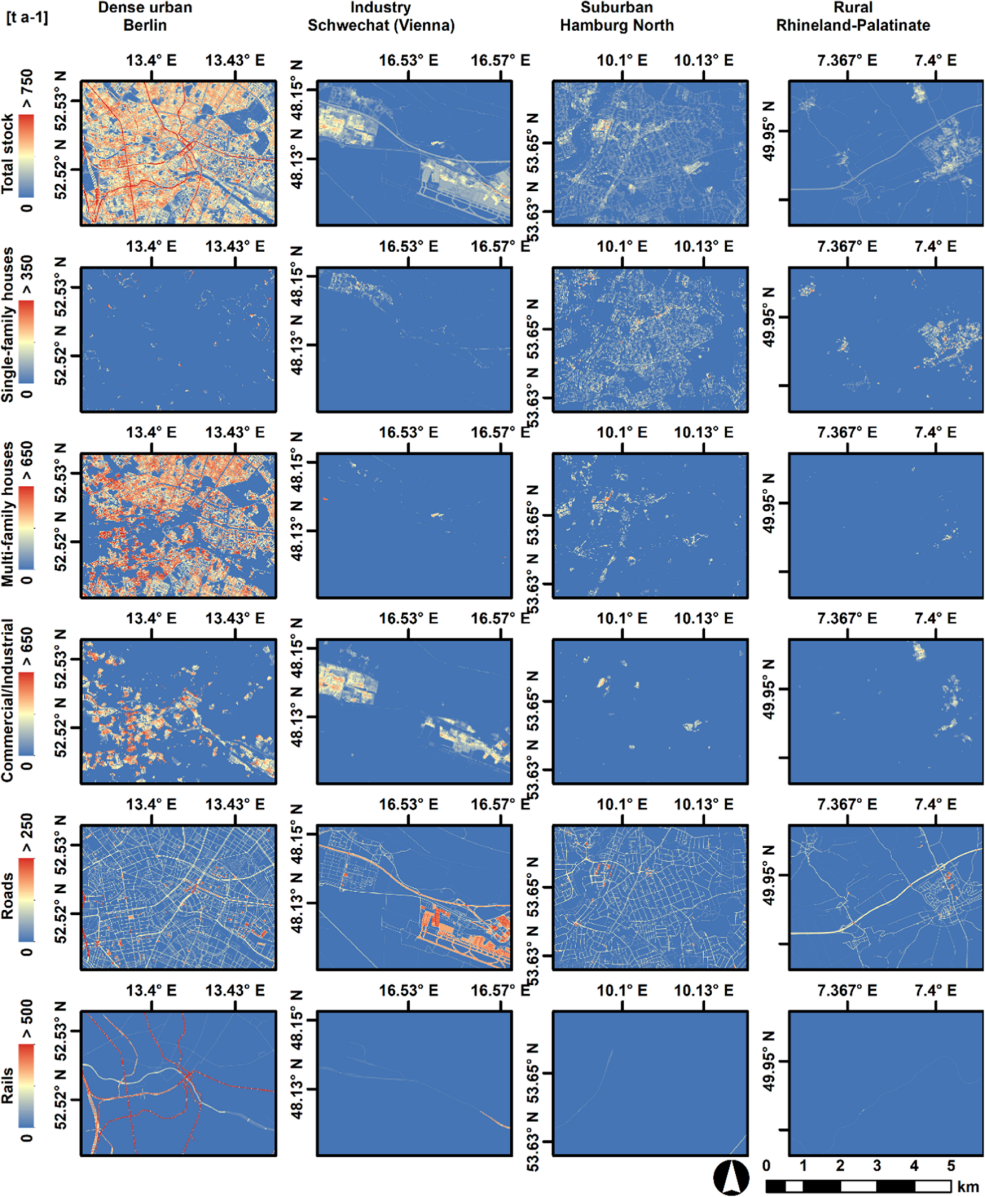
Close-ups of material stock maps (10 m resolution) in four locations in Germany (columns 1, 3, 4) and Austria (column 2), showing total stocks (first row) and different specific structures (rows 2–6) for the same location. Note the varying scales across rows.

Figure 3 shows examples of locations with very different densities and structures of material stocks, three in DE and one in AT. Column 1 depicts a dense urban setting (Berlin), column 2 depicts a site dominated by industry and transport infrastructures showing the refinery Schwechat in the upper-left and Vienna’s airport located in Lower Austria in the lower- right part, column 3 depicts a suburban setting in the north of Hamburg, and column 4 depicts a rural setting in Rhineland-Palatinate, DE. The rows demonstrate the thematic richness of the dataset by displaying the layers of single-family houses, multifamily houses, commercial and industrial buildings, roads, and railroads. As expected, some structures are sparse or nonexistent in specific locations, e.g., there are few single-family houses in central Berlin and few, if any, railroads in the chosen rural and suburban settings.

**Figure 4:**
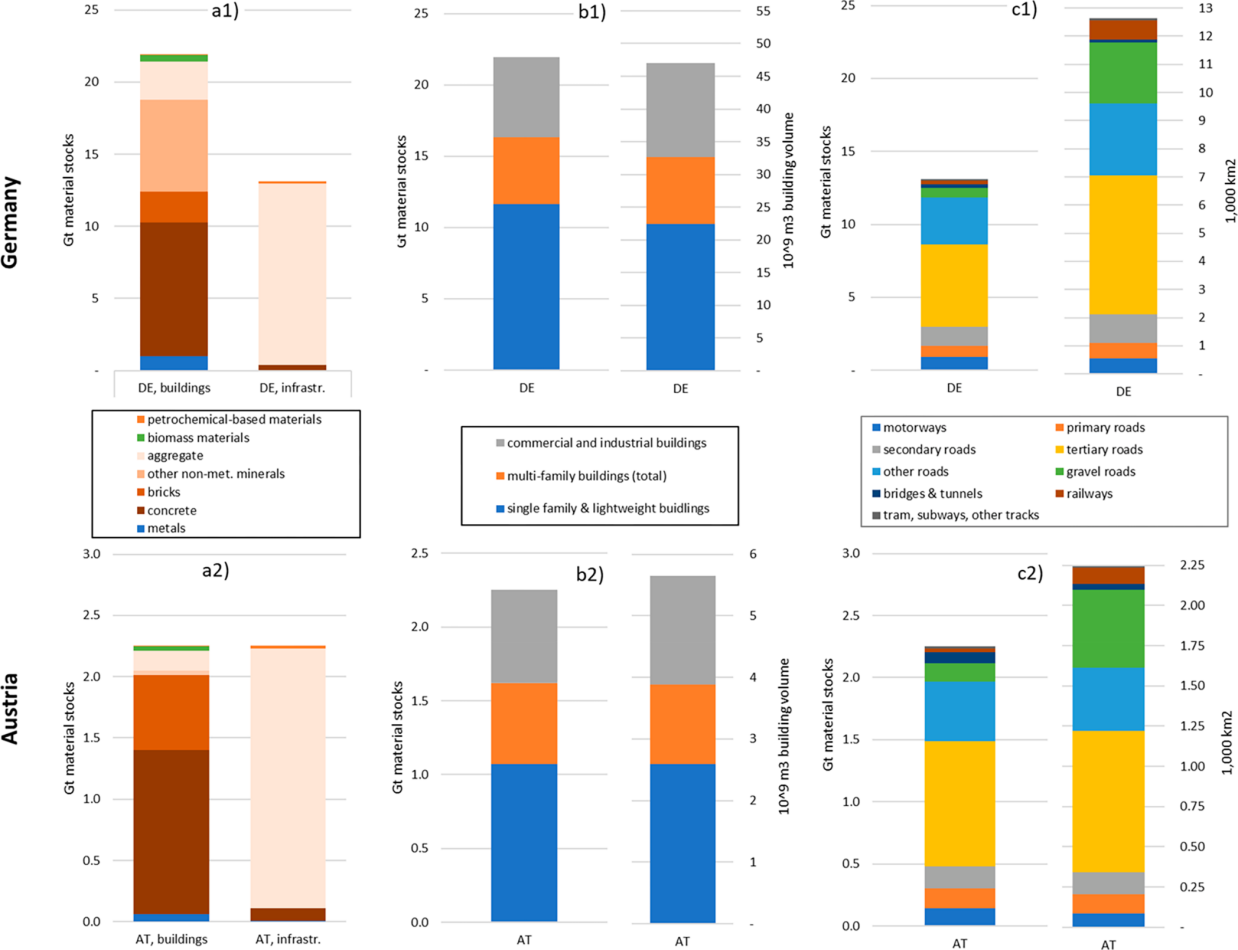
Material stocks and other physical dimensions of buildings and infrastructures in Germany (a1–c1) and Austria (b1–c1). First column: material stocks in buildings and infrastructures broken down by seven major categories of materials in Germany (a1) and Austria (a2). Second column: material stocks (left bar) and building volume (right bar) of buildings in Germany (b1) and Austria (b2) broken down by three major types of buildings. Third column: Material stocks (left bar) and surface area (right bar) of different types of infrastructures (seven types of roads, bridges, and tunnels, and two types of rail infrastructure) in Germany (c1) and Austria (c2).

Figure 4 summarizes aggregate material stock results for DE (top row, a-c1) and AT (lower row, a-c2). In DE, the mass of infrastructures is substantially lower than that of buildings, whereas in AT the mass of infrastructures is similar to those of buildings. Concrete and other nonmetallic minerals account for the largest part of the total mass, while metals, biomass (mostly timber), and other materials play a smaller role (first column, a1-2). Single-family houses make up roughly half of the material stocks and volume of all buildings in both countries. Multifamily houses and industrial/commercial buildings account for the rest and are of similar magnitude. Roads by far surpass rail infrastructures in terms of both area and mass, with smaller (tertiary and gravel roads) playing a substantial role, in particular in terms of length but also in terms of mass.

## 4 Discussion

### 4.1 Spatial Resolution

**Figure 5:**
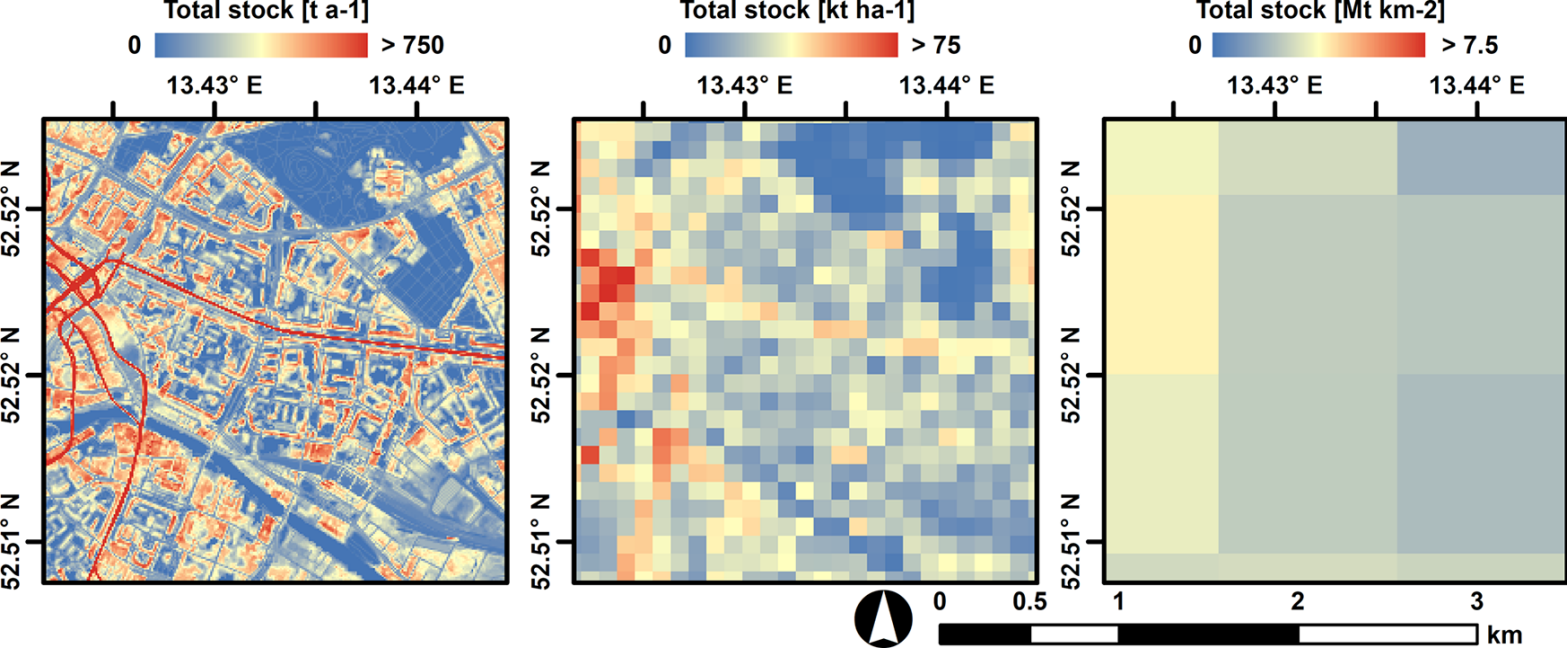
Comparison of maps of central Berlin with different resolutions: left, 10 m; middle, 100 m; right, 1 km. Many current material stock maps are at 1 km resolution. While high-resolution maps reveal more spatial detail, uncertainties and imprecisions of material stock results are lower at coarser resolutions where data artifacts or methodological assumptions are removed during data aggregation.

Due to the resolution of the S1 and S2 data and the characteristics of OSM data, we can display results at 10 m spatial resolution (Figure 5), which allows us to finely distinguish different structures (Figures 3 and 5). In contrast, many published material stock maps and building volume predictions (e.g., ref (79)) have scaled-down resolutions of 1 km (as in the right plate in Figure 5), though some local examples present the cadaster level.^32^ NTL products usually have a resolution of 500–750 m;^42,43,48,80,81^ results are often aggregated to even larger units. NTL are widely used to downscale economic measures such as economic activity,^82^ poverty^83^ or asset wealth^84^ to small spatial scales on which no data are available from census statistics. Given the much higher spatial resolution of our data, as well as their ability to identify objects associated with specific functions or services^85,86^ that allow causal inferences, we assume that they could be a basis for more accurate, respectively higher resolved, downscaling methods. Some types of imprecisions resulting, e.g., from processing algorithms or statistical variation, problems in the input data (e.g., possible inconsistencies of sentinel data and OSM), or data manipulation (i.e., “noise”) may be canceled out when aggregating to coarser resolutions such as 100 or 1000 m (as used in many current datasets). Most prominently, we used an empirical correction factor of 0.53 when converting built-up area to building area, as neither EO nor OSM data could properly account for the roof overhang and smaller flat infrastructures. While preserving spatial detail, which allows for comparative interpretation of highly detailed spatial patterns, this factor introduces an error at the 10 m scale because each 100 m^2^ pixel has a maximum building area of 53 m^2^. While the distinction between built-up land (buildings plus infrastructure) and other land is accurate, as it is not affected by that approach, the classification of built-up land as either building or infrastructure is blurred at the pixel level. Hence, data on the mass of material stocks will likely be more robust at coarser scales, in particular when differentiating specific materials is of interest. Note, however, that any aggregation may suffer from the modifiable areal unit problem, i.e., different results of the aggregation depend on the boundaries of the coarser resolution grid, which can bias statistical analyses.^87^ We are convinced, though, that a spatial resolution of input data of 10 m is useful. For example, the entire calculation of infrastructure-related stocks depends on fine-scale information that is able to capture the related linear and largely narrow features (see Figure 1, left-hand side workflow). Our aggregation approach accordingly targets a spatial resolution that represents the optimal compromise between a smooth map representation of stocks at landscape to national scales, while preserving a sensible level of detail.

### 4.2 Comparison of Results against Statistical Data

The mapped material stock results measured in different dimensions were cross-checked against statistical data sources. A comparison of the length of infrastructures as reported in OSM with statistical sources reveals that OSM data yield higher results regarding the length of road and railway networks than statistical sources (SI, Figures SI_5–SI_7). This is consistent with a tendency toward under-reporting of infrastructure length in statistical sources,^65^ which results from the lack of harmonization of reporting between different owners or managers of infrastructures (federal, state, communal, and private owners) and lacking coverage of minor roads, gravel roads, footpaths, and similar structures. OSM data are generally assumed to be robust for countries like AT and DE;^88,89^ hence, we assume that OSM-based results are likely more comprehensive than those of statistical reports.

Comparing building areas and volumes between values calculated from Austrian building statistics^90^ and mapping results reveals that our results for building area are higher than those calculated from statistical data (SI, Section S5). Differences can be observed both in building areas and building volumes. The comparison of the building volumes for DE shows that our estimates are on average 41% larger than those derived from statistical data. For AT, we found even larger differences when comparing building volumes derived from an extrapolation of AT building statistics (SI) and data from Lederer et al.:^53^ our estimates of building volumes were 107% larger than those extrapolated from statistics and our estimates of gross footprint area exceeded data from statistics by 30–104%. A previous comparison of results derived with 3D models for Vienna with statistical data found similar differences.^53^ Reasons included lacking inclusion of flats in rooftops in statistical data, differences in definitions between mapped building volumes and statistical data (e.g., differences in the calculation of roof volumes), and a general tendency of under-reporting in statistical data. All of this is also relevant for explaining the difference between our results and statistical data; additional reasons are the lacking coverage of some building types in statistics, differences in the definition of building volumes and buildings established after the last available census for Austria that refers to the year 2011.

**Figure 6:**
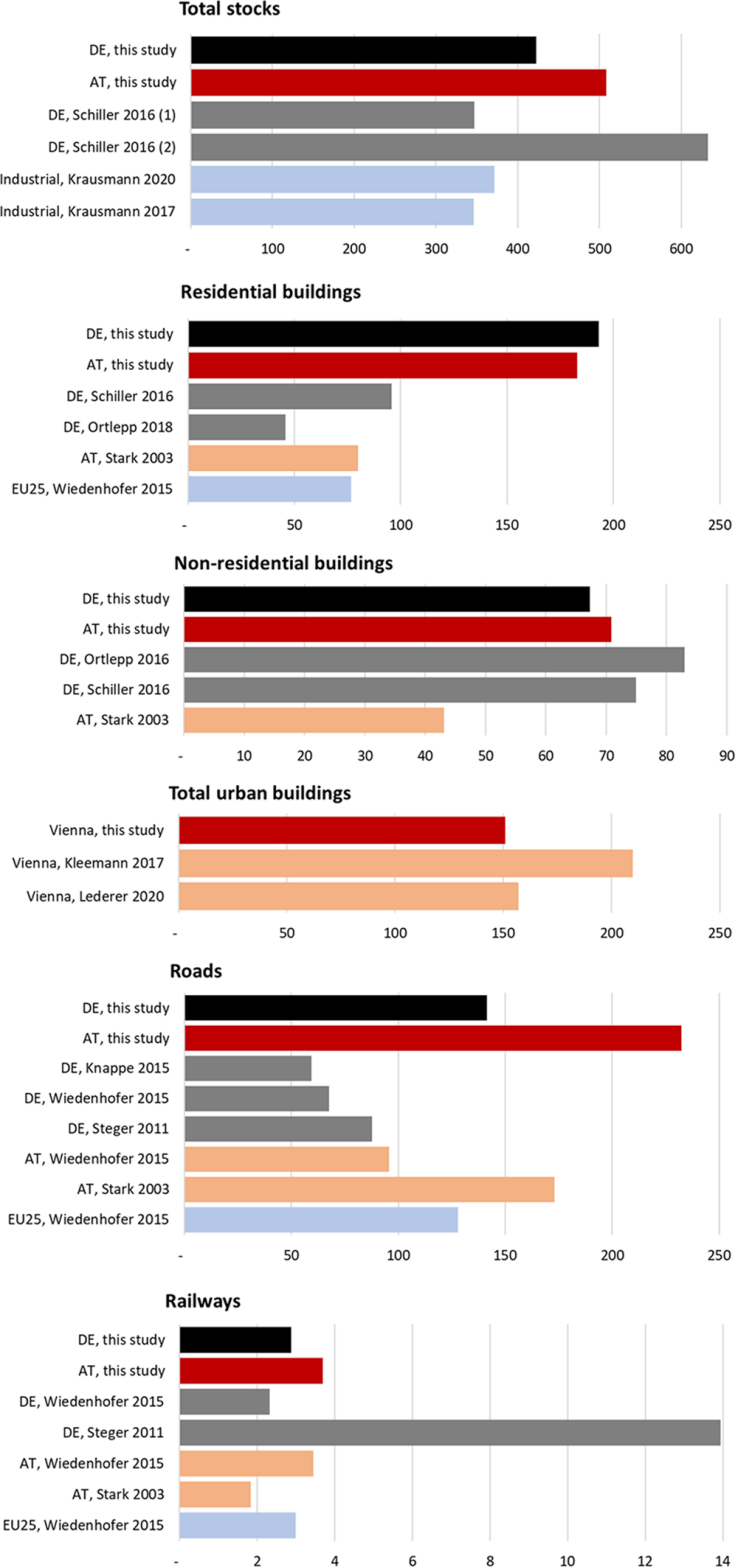
Comparison of our results regarding the mass of material stocks with data from the literature, expressed as tons of total stocks per capita of population. Sources: DE, Schiller 2016 (1) (bottom-up);^51^ DE, Schiller 2016 (2) (top-down);^51^ Industrial Krausmann 2017^12^ (industrialized countries excl. China); DE, Ortlepp 2016;^50^ DE, Ortlepp 2018;^92^ AT, Stark 2003;^91^ EU25, Wiedenhofer 2015;^65^ Vienna, Kleemann 2017;^39^ Vienna, Lederer 2021;^53^ DE, Wiedenhofer 2015;^65^ AT, Wiedenhofer 2015;^65^ DE, Knappe;^66^ DE, Steger 2011.^64^

Comparing the results for the mass of buildings and infrastructures with previous estimates from the literature shows good agreement for some stock types, but generally higher overall mass in our mapping (Figure 6). With respect to total stocks, our estimates for Austria (∼540 t/cap) and Germany (∼450 t/cap) are within the range spanned by the high dynamic top-down (inflow-driven) and low stock-driven bottom-up estimates for Germany of a previous study.^51^ They are higher than the results of an inflow-driven model for the entire “industrial” region^12^ (first part of Figure 6). While our results for nonresidential (commercial and industrial) buildings are similar to previous estimates (third part of Figure 6), we find substantially higher material stocks in residential buildings than previous estimates^51,65,66,91,92^ (second part of Figure 6). Results for roads are larger than those of previous studies,^64,65^ which is most likely related to the tendency of previous studies to underestimate minor roads, for which data quality is poor (fourth part of Figure 6). Railroad results fit well with previous estimates,^65^ except for those derived by Steger et al.,^64^ who included service tracks and stations (fifth part of Figure 6). A comparison of our results for buildings in Vienna with those calculated in previous GIS-based studies^39,53^ revealed that our material stock results are lower than those found in earlier works, in particular in ref (39) (see SI, Figure SI_13). The main reason for this deviation is that this older study^39^ had used a different dataset for material intensities. The dataset used here, developed by the same authors as in the studies mentioned,^39^ is not only much larger (207 instead of 66 buildings), but also shows generally lower average material intensities, as also visible in the much better fit with new work of that group.^53^

Our results are the first derived from fine-scale satellite data in a “wall-to-wall” fashion and very well align with the spatially explicit distribution of 3D-features in the lidar-based ground truth (also see Figure SI_4 and Table SI_1). We are hence confident that our assessment is robust enough to suggest that previous estimates are probably too low, mostly because our results rely on satellite and crowd-sourcing data that can detect structures not included or underestimated in statistical surveys. However, we cannot entirely rule out the possibility of overestimations that might result from misclassifications, not fully representative MI factors, or other data errors. More research studies are certainly warranted to corroborate and refine these results, which shall become feasible with an increasing number of high-resolution reference datasets expected to be released in the future.

### 4.3 Outlook

Spatial structures of buildings and infrastructures have important implications for societies’ resource requirements.^27,93−96^ Thematically and spatially highly resolved maps such as those presented here can help in analyzing the spatial dimensions of the stock-flow-service nexus,^6,29^ thereby helping to reduce resource use without impairing delivery of crucial services and well-being contributions.^97^ This includes, for example, resource-sparing development of urban form,^27^ provision of information for estimating secondary resource potentials for reuse and recycling from end-of-life stocks.^20^ This novel method can also provide an improved high-resolution mapping to investigate urbanization, urban form, and infrastructure developments and inform infrastructure vulnerability studies.^36^

Although material stock maps derived from cadastral data are superior in terms of data accuracy when aiming to establish resource cadasters for secondary resources and closing material cycles in individual cities,^32,33,35,52^ the maps presented here can close the gap between rather coarse material stock estimates (usually ∼1 km spatial resolution) covering large areas that can be derived from NTL^46,48^ and highly detailed cadaster-based studies limited to small areas. The method presented here is based on freely and openly available data, can be extended over national areas and replicated over any time span for which satellite and crowd-sourcing data, training data for assessing the height of buildings, and robust MI factors are available. Because S1 and S2 data are collected continuously, future studies could provide maps annually and allow for fine-grained change detection.^22^ The European Commission is currently preparing for annual, global maps of built-up areas and population to be produced in the Copernicus services; we hope that this study can contribute to these products. The method presented here could hence help to monitor the development of societies’ material stocks over large areas and time in a spatially highly resolved manner.

## Supporting Information Available

Earth observation products; mapping infrastructures using Open Street Map (OSM) data; material intensities of buildings and infrastructure; comparison of mapped building volumes with statistical data; additional results: detailed material stock data for Germany and Austria (PDF)
Excel spreadsheet containing four tables reporting MI factors for AT and DE as well as additional results for AT and DE (XLSX)
