# Supplementary material for: High-Resolution Maps of Material Stocks in Buildings and Infrastructures in Austria and Germany
Source: Environ Sci Technol. 2021 Feb 18;55(5):3368–79. doi: 10.1021/acs.est.0c05642 (PMC7931449; doi:10.1021/acs.est.0c05642)
Supplement: Supplementary file 1 — es0c05642_si_001.pdf [file es0c05642_si_001.pdf]

SUPPLEMENTARY INFORMATION:
High-resolution maps of material stocks in buildings and infrastructures in Austria and Germany

Helmut Haberl, Dominik Wiedenhofer, Franz Schug, David Frantz, Doris Virág, Christoph Plutzar, Karin Gruhler, Jakob Lederer, Georg Schiller, Tomer Fishman, Maud Lanau, Andreas Gattringer, Thomas Kemper, Gang Liu, Hiroki Tanikawa, Sebastian van der Linden, Patrick Hostert

Contents

In this supplementary information, we provide a documentation of the definitions and procedures applied. The document proceeds by introducing the data sources, the rationale of processing and all specific steps taken, and then documents the various cross-checks and validations that were conducted. Finally, we provide additional results. 25 pages, 13 Figures, 9 Tables

Table of contents

1. Earth observation products 2
   - 1.1 Built-up area 2
   - 1.2 Building area 2
   - 1.3 Building height validation 4
   - 1.4 Building types 6

2. Mapping infrastructures using Open Street Map (OSM) data 8
   - 2.1 Data extraction and aggregation 8
   - 2.2 Robustness of OSM data 12

3. Material intensities of buildings and infrastructure 13
   - 3.1 Definition of volumes used to estimate material intensities 13
   - 3.2 Material intensity factors 14

4. Comparison of mapped building volumes with statistical data 16
   - 5.1 Building volumes in Germany 16
   - 5.2 Building volumes in Austria 19

5. Additional results: Detailed material stock data for Germany and Austria 22

1. Earth observation products

1.1 Built-up area

A systematic validation of sub-pixel land cover shares has been established in Schug et al.[1], using 160 validation sites with a total of 36,000 sampling points across Germany and Austria. In this study, radar Sentinel-1 time series were used to complement optical Sentinel-2 data, which especially helped to reduce commission errors in areas with seasonal soil occurrence. In addition, the Tasseled Cap Greenness component (standard deviation and 90th percentile) was used instead of NDVI statistics to improve results in densely built-up urban agglomerations where NDVI is affected by a shadow effect in street canyons. Figure SI_1 shows validation results for the built-up area for the 160 validation sites, with an RMSE of 19%, MAE of 13%, and R^2 of 0.74 and slope of 0.75. The validation was performed at a spatial resolution of 20m, as a validation on the native 10 m resolution would be affected by the absolute geolocation error of Sentinel (10-12 m) and reference Google Earth imagery (unknown geolocation error).

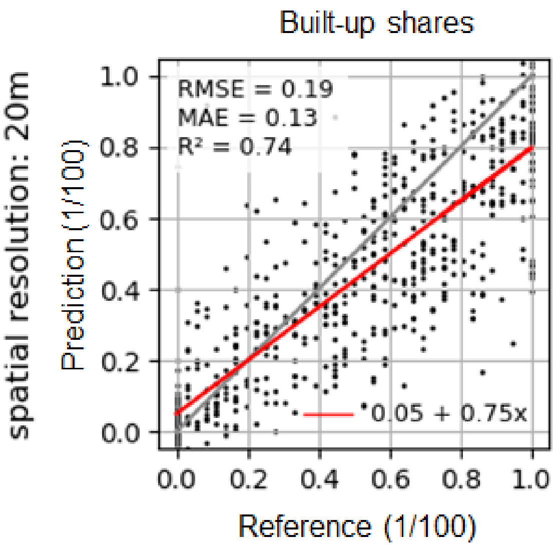

Figure SI_1. Updated validation for sub-pixel built-up share mapping. Validation based on the framework presented in Schug et al.[1]. RMSE: Root Mean Squared Error, MAE = Mean Absolute Error.

1.2 Building area

This study applies a correction factor of 0.53 to building density maps that accounts for roof overhang and smaller infrastructure not covered by the OSM-based infrastructure layer. This factor is based on empirical findings. We compared building density mapping results with

rasterized cadastral building footprint data provided by the Berlin Senate for Urban Development and Housing at different aggregation levels, which is available at the following URL: https://www.stadtentwicklung.berlin.de/umwelt/umweltatlas/i610.htm.

Figure SI_2 illustrates that built-up area maps overestimate actual building area. With decreased spatial resolution, the surface overestimation becomes linear, resulting in a correction factor of about 0.53 based on slope, at 1000 m resolution. Applying this constant factor at a spatial resolution of 10 m, resulting building area estimates are not completely accurate; see discussion. However, this method ensures that highly detailed spatial patterns remain, and guarantees that absolute values at larger aggregation levels become more accurate.

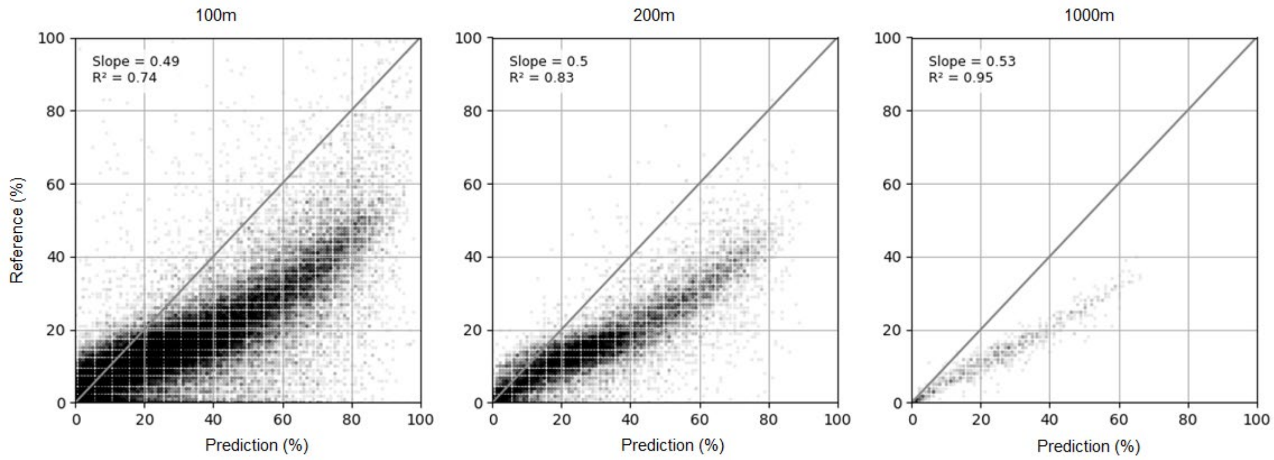

Figure SI_2. Building area correction factor from predicted built-up area and reference building area derived from the Berlin Senate for Urban Development and Housing. Overestimation is due to roof overhang and impervious features unaccounted for in the OpenStreetMap.

Lightweight buildings like garages are substantially lighter than residential buildings. However, from Sentinel data alone, it is impossible to separate garages that are attached to buildings, and an analysis of the well-attributed 3D building model of North Rhine Westphalia (see Table_SI_1 for data source) revealed that this building type covers a high share of pixels (10%; see Figure SI_3 left), which we have classified as single-family houses. Therefore, we used a correction factor to reduce the building area of single-family pixels by 10%, which is then added to the lightweight building area. For computing the volume of the garages within the lightweight category, we further used a constant height of 2.7 m, which was derived as the median garage height from the 3D building model analysis (Figure SI_3 right).

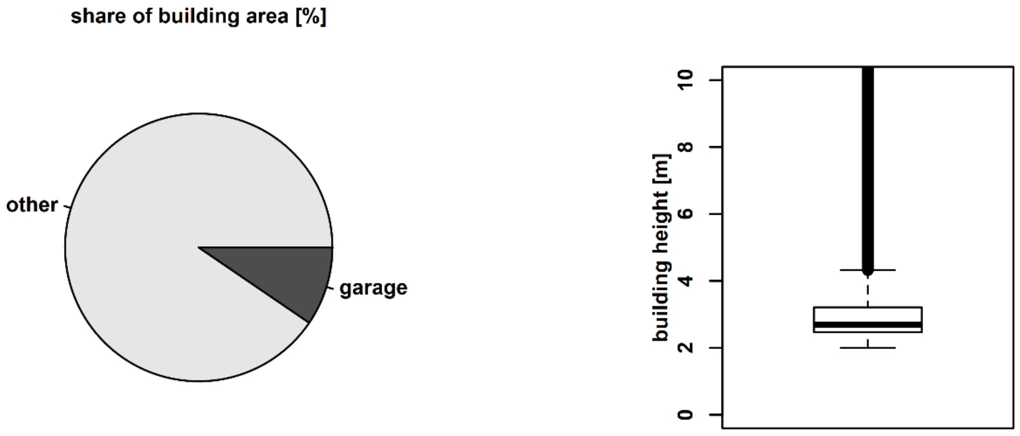

Figure SI_3. Share of building area covered by garages (left) and boxplot of garage height (right) in our single-family house class as derived from an analysis of the 3D building model for the state North Rhine Westphalia, Germany.

1.3 Building height validation

A detailed validation of the building height product for Germany is available in Frantz et al.[2]. Figure SI_4 presents an additional validation for Vienna. The validation is based on a stratified sampling scheme where the same number of validation samples was collected for each meter of height, whenever possible. Ordinary least squares (OLS) regression provides a good estimation for the model's capability to predict buildings of different height classes. The Root Mean Square Error (RMSE) based on this sample estimates the height class uncertainty. However, in relative terms, there are few high-rise buildings in Germany and Austria. Therefore, the OLS estimate is skewed towards higher building heights. Consequently, weighted least squares (WLS) regression was employed to weight each building height class with the frequency of its occurrence in the corresponding reference dataset. The WLS estimate is more representative of the areal accuracy, e.g. when reporting a mean building height for a given area (e.g. a city, district or state). Accordingly, the weighted RMSE is a measure of the areal height uncertainty. Complementary to this, OLS and WLS regression through the origin are reported as building height is a parameter with a well-defined lower boundary. The reference data used in Frantz et al.[2] and Figure SI_1 are listed in the table below.

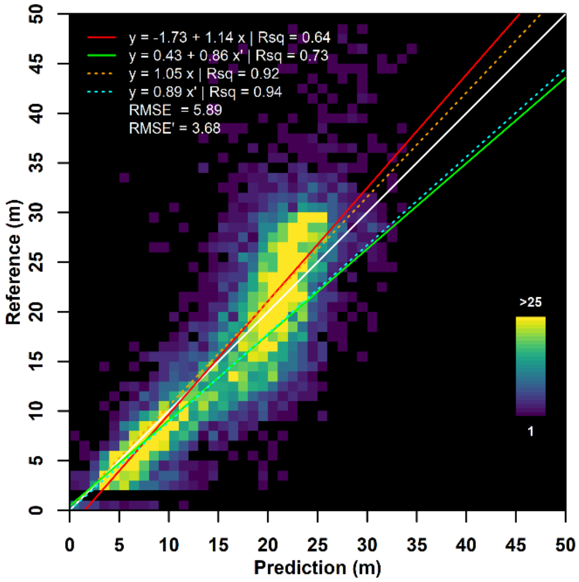

Figure SI_4. Extended building height validation for testing model extrapolation and transferability. The reference data were obtained from the 3D Building Model of Vienna; the original validation for five German sites is presented in Frantz et al.[2]; see Table SI_1 for the reference datasets used. White Line = one-to-one; red line: ordinary least squares regression, orange line: ordinary least squares regression through origin; green line: weighted least squares regression; cyan line: weighted least squares regression through origin; RMSE: Root Mean Squared Error, RMSE' = weighted RMSE; weights were obtained from the frequency of occurrence within the reference dataset.

Table SI_1. References of building data used for validation of building data.

Site	Data provider	License	URL
Berlin	Berlin Partner für Wirtschaft und Technologie GmbH	Custom license: https://www.businesslocationcenter.de/berlin3d-downloadportal/documents/terms.en.html	https://www.businesslocationcenter.de/en/economic-atlas/download-portal/
Hamburg	Freie und Hansestadt Hamburg, Landesbetrieb Geoinformation und Vermessung	Data licence Germany – attribution – version 2.0	http://suche.transparenz.hamburg.de/dataset/3d-stadtmodell-lod2-de-hamburg4?forceWeb=true
Potsdam	Landeshauptstadt Potsdam (LHP)	Unspecified open data license	https://opendata.potsdam.de/explore/dataset/3d-gebaudemodell-lod2-citygml/information
North Rhine West-phalia	Bezirksregierung Köln, Geobasis NRW	Data licence Germany – attribution – version 2.0	https://www.bezreg-koeln.nrw.de/brk_internet/geobasis/3d_gebaeudemodelle/index.html
Thuringia	Kompetenzzentrum Geodateninfrastruktur Thüringen (GDI-Th)	Data licence Germany – attribution – version 2.0	https://www.geoportal-th.de/de-de/Downloadbereiche/Download-Offene-Geodaten-Th%C3%BCringen/Download-3D-Geb%C3%A4ude
Vienna	Stadt Wien – https://data.wien.gv.at	Creative Commons Namensnennung 4.0 International	https://www.data.gv.at/katalog/dataset/86d88cae-ad97-4476-bae5-73488a12776d

1.4 Building types

Five building types have been mapped at a spatial resolution of 10 m: Commercial and industrial buildings, light-weight buildings, single-family residential buildings, multi-family residential buildings and high-rise buildings. The former four classes were derived based on a random forest classification procedure established in Schug et al.[3]. High-rise buildings were distinguished from multi-family residential buildings using a height threshold of 30 m, as high-rise buildings have particular characteristics with regard to material stocks. The classification was validated for Germany with an overall accuracy of 81.4 %[3]. Additional training data at manually selected sites was collected in Austria to extend the previously used model. A separate validation for Austria was conducted based on a stratified random sampling with 15 samples for commercial and industrial buildings, single-family and multi-family residential buildings and 10 samples for

light-weight buildings per federal state (nine states). Samples that were placed on surfaces that were erroneously identified as building in the building density product were excluded, resulting in a total of 427 validation sites and an overall accuracy of 83.6 % (Table SI_2).

Table SI_2: Error matrix of the building type classification in Austria. IC = Industrial and Commercial Buildings, SF = Single-Family Housing, MF = Multi-Family Housing, LS = Light-Weight Buildings, OA = Overall Accuracy

	Reference						
		**IC**	**SF**	**LB**	**MF**	**n**	**User's Acc.**
**Prediction**	**IC**	108	2	0	7	**117**	92.30 %
	**SF**	4	116	1	5	**126**	92.06 %
	**LB**	2	31	45	0	**78**	57.69 %
	**MF**	10	7	1	88	**106**	83.01 %
	**n**	**124**	**156**	**47**	**100**	**427**	
	**Prod. Acc.**	87.09 %	74.35 %	95.74 %	88.00 %		
	**OA**	83.60 %					

2. Mapping infrastructures using Open Street Map (OSM) data

2.1 Data extraction and aggregation

OSM data were taken from https://download.geofabrik.de/index.html (date of access: 30.01.2020). Using Osmium (https://osmcode.org/osmium-tool/), the following layers were extracted for Austria and Germany separately:

1. Key = highway, value = *: lines (Austria 1,867,492 features, Germany 11,463,386 features)
2. Key = railway, value = *: lines (Austria 50,716 features, Germany 317,896 features)
3. Key = aeroway, value = taxiway: lines (Austria 630 features, Germany 5984 features)
4. Key = aeroway, value = runway: lines (Austria 83 features, Germany 1074 features)
5. Key = aeroway, value = apron: multipolygons (Austria 83 features, Germany 1074 features)
6. Key = amenity, value = parking: multipolygons (Austria 53,366 features, Germany 391,332 features)
7. Key = railway, value = platform, value = subway: lines (Austria 182 features, Germany 48 features); multipolygons (Austria 4 features, Germany 711 features)

In OSM, 32 types of roads are distinguished. We aggregated them to 8 groups (motorways, primary, secondary and tertiary roads, gravel roads, other roads, and "zero"). The last category "zero" refers to roads for which we do not assume the existence of human-made material stocks.

Table SI_3 Aggregation of OSM road categories to the groups of road infrastructures used for the material stock calculcation.

MI-cluster	OSM-category	OSM description taken from: https://wiki.openstreetmap.org/wiki/Map_Features)
motorway	motorway	A restricted access major divided highway, normally with 2 or more running lanes plus emergency hard shoulder. Equivalent to the Freeway, Autobahn, etc.
motorway	motorway_link	The link roads (sliproads/ramps) leading to/from a motorway from/to a motorway or lower class highway. Normally with the same motorway restrictions.
primary	trunk	The most important roads in a country's system that aren't motorways.
primary	trunk_link	The link roads (sliproads/ramps) leading to/from a trunk road from/to a trunk road or lower class highway.
primary	primary	The next most important roads in a country's system. (Often link larger towns.)
primary	primary_link	The link roads (sliproads/ramps) leading to/from a primary road from/to a primary road or lower class highway.
secondary	secondary	The next most important roads in a country's system. (Often link towns.)
secondary	secondary_link	The link roads (sliproads/ramps) leading to/from a secondary road from/to a secondary road or lower class highway.
tertiary	tertiary	The next most important roads in a country's system. (Often link smaller towns and villages)
tertiary	tertiary_link	The link roads (sliproads/ramps) leading to/from a tertiary road from/to a tertiary road or lower class highway.
tertiary	unclassified	The least important through roads in a country's system – i.e. minor roads of a lower classification than tertiary, but which serve a purpose other than access to properties. (Often link villages and hamlets.)
tertiary	residential	Roads which serve as an access to housing, without function of connecting settlements. Often lined with housing.
tertiary	living_street	For living streets, which are residential streets where pedestrians have legal priority over cars, speeds are kept very low and where children are allowed to play on the street.
other	service	For access roads to, or within an industrial estate, camp site, business-park, car-park, alleys, etc.
other	track (grade1)	Roads for mostly agricultural or forestry uses. Solid, usually a paved or sealed surface.
gravel	track (grade2)	Roads for mostly agricultural or forestry uses. Solid but unpaved, usually an unpaved track with surface of gravel.
gravel	track (grade3)	Roads for mostly agricultural or forestry uses. Mostly solid. Even mixture of hard and soft materials. Almost always an unpaved track.
zero	track (grade4)	Roads for mostly agricultural or forestry uses. Mostly soft. Almost always an unpaved track prominently with soil/sand/grass, but with some hard or compacted materials mixed in.
zero	track (grade5)	Roads for mostly agricultural or forestry uses. Soft. Almost always an unimproved track lacking hard materials, same as surrounding soil.

zero	track (no info)	Roads for mostly agricultural or forestry uses. If no tracktype tag is present, the track is rendered with a dot-dash line style (as shown right).
zero	path	A non-specific path.
other	footway	For designated footpaths; i.e., mainly/exclusively for pedestrians. This includes walking tracks and gravel paths.
other	cycleway	For designated cycleways.
zero	bridleway	For horse riders.
other	steps	For flights of steps (stairs) on footways.
other	pedestrian	For roads used mainly/exclusively for pedestrians in shopping and some residential areas which may allow access by motorised vehicles only for very limited periods of the day.
zero	construction	For roads under construction.
motorway	raceway	A course or track for (motor) racing.
other	rest area	Place where drivers can leave the road to rest, but not refuel.
other	road	A road/way/street/motorway/etc. of unknown type. It can stand for anything ranging from a footpath to a motorway.
other	services	A service station to get food and eat something, often found at motorways.
other	platform	A platform at a bus stop or station.

Road data extracted from OSM and used in the calculation of material stocks is summarized in Table SI_4. The buffer width was derived based on expert evaluations for calculating area estimates based on line buffering for Germany[4]; the data we used in the calculations are reported in the last column. The data also include information on bridges and tunnels which we also used to calculate their respective masses using MI factors explained below.

Table SI_4 Descriptive statistics for 32 road categories extracted from OSM for Austria and Germany, and width information (buffer) used in the calculations

Category	count	total length [km]	bridge length [km]	tunnel length [km]	width mean [m]	width count	buffer used [m]
Motorway	78856	30532	1540	540	9.5	3057	12.0
motorway_link	47244	6348	211	20	5.9	609	6.5
Primary	243020	49018	820	173	8.1	4015	5.5
primary_link	20821	1446	26	3	5.9	185	5.5
Trunk	31303	7359	460	178	9.3	805	10.0
trunk_link	19560	2105	84	8	6.4	228	6.5
Secondary	433382	109192	879	92	6.5	8215	5.5
secondary_link	10881	539	6	1	4.7	85	5.5
Tertiary	399883	124387	625	52	5.6	10746	4.5
tertiary_link	5147	236	2	-	5.1	49	4.5
Unclassified	437712	179164	531	61	3.9	23277	4.5
Residential	1970023	351749	558	60	4.2	68548	4.5
living_street	134749	15461	11	2	4.1	4455	4.5
Service	2929171	243132	398	458	3.4	39983	2.5
track (grade1)	418638	155193	356	62	2.9	33692	2.5
track (grade2)	613476	308013	173	35	2.8	56084	2.5
track (grade3)	654708	286917	107	17	2.5	41678	2.0
track (grade4)	518770	180093	45	7	2.3	35523	2.0
track (grade5)	400121	114539	49	3	2.2	22817	2.0
track (no info)	675450	257863	172	53	2.5	8467	2.0
Path	1297494	247860	1056	190	1.4	134227	1.0
Footway	1571092	109311	788	455	1.9	58006	1.5
Cycleway	119001	34292	362	51	2.3	9342	1.5
Bridleway	7213	3068	1	-	1.8	597	1.5
Steps	220426	3130	18	49	2.3	15800	1.5
Pedestrian	30577	2033	14	9	5.5	1148	4.0
Construction	8673	1816	56	107	4.6	189	3.0
Raceway	1494	564	3	-	10.8	103	7.5
rest_area	1071	539	-	-	-	-	6.0
Road	2415	468	2	1	5.9	52	4.0
Services	311	303	-	-	-	-	6.0
Platform	13803	272	-	-	1.7	44	1.5

Data on railways in OSM exist for ten types of railways, which includes tram, subway and other rail bound transport infrastructures. These data also allow calculations of bridge and tunnel lengths, as shown in Table SI_5.

Table SI_5. Descriptive statistics of railway data extracted from OSM for Austria and Germany.

**Category**	**count**	**total length [km]**	**bridge length [km]**	**tunnel length [km]**	**width mean [m]**	**width count**	**buffer used [m]**
Rail	235406	94546	1495	1573	1962.04	36	6.0
Abandoned	40766	18757	97	79	2.70	2382	2.0
Disused	21452	6131	95	38	3.03	19	2.0
Tram	23168	5603	78	94	8.27	11	3.5
light_rail	8666	2257	69	245	-	-	3.5
Subway	5592	1453	74	924	-	-	4.0
narrow_gauge	6184	1667	17	27	1.30	1	3.5
Preserved	1860	988	7	3	2.06	1	3.5
Platform	9541	830	3	8	2.70	166	2.0
Construction	1196	881	16	481	13.00	2	8.5

2.2 Robustness of OSM data

Substantial differences between official statistical data sources, reports and spatially-explicit information are a well-known problem for infrastructures. The main reason is that statistical data on road and rail networks notoriously suffers from lacking harmonization of definitions between different owners and managers of infrastructure such as federal, state, communal and private operators. In Figures SI_5 and SI_6 we compare OSM-derived data on road and railway networks against statistical data compiled in previous work, as well as information from national statistical agencies. We find that road and railway network lengths match reasonably well with official statistics as far as high-level infrastructures are concerned, but lower-rank infrastructures are missing or underreported in some sources. Please note that information for roads from DESTATIS has known problems with substantial under-reporting of residential and tertiary communal roads, for which an estimate has been added in modelling studies[5]. For rails, only information of the public-owned network is available.

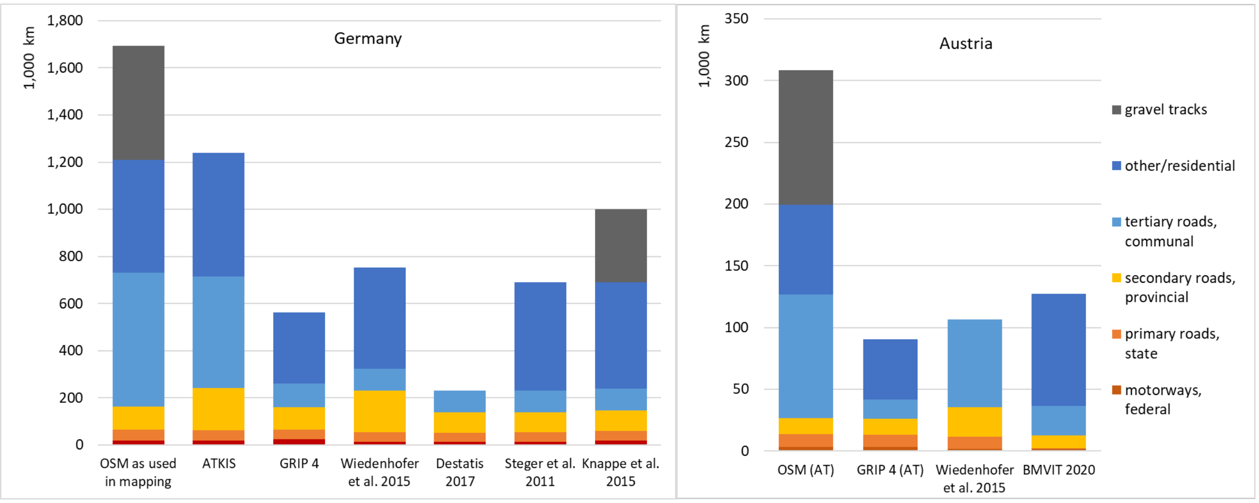

Figure SI_5. Comparison of road length data from OSM versus official statistics. References: OSM data as used in the mapping, see above; ATKIS[6]; GRIP 4[7]; Wiedenhofer et al. 2015[5]; Destatis 2017[8]; Steger et al. 2011[9]; Knappe et al. 2015[10] BMVIT 2000[11]

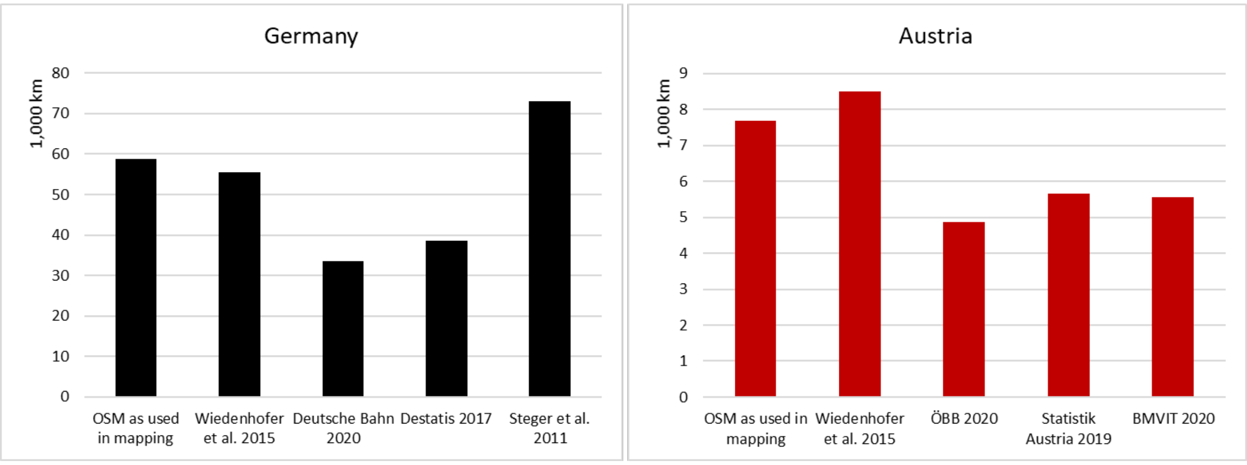

Figure SI_6. Comparison of railway length data from OSM versus official statistics. Sources: OSM extracted and used in this study (see above); Wiedenhofer et al. 2015 [5]; Deutsche Bahn 2020 [12]; Destatis 2020 [8]; Steger et al. 2011 [9]; ÖBB 2020 [13]; Statistik Austria 2019 [14]; BMVIT 2020 [11]

The subway network in OSM shows slightly (15%) more length compared to data from Lederer et al.[15], which is due to inclusion of side tracks (Figure SI_7).

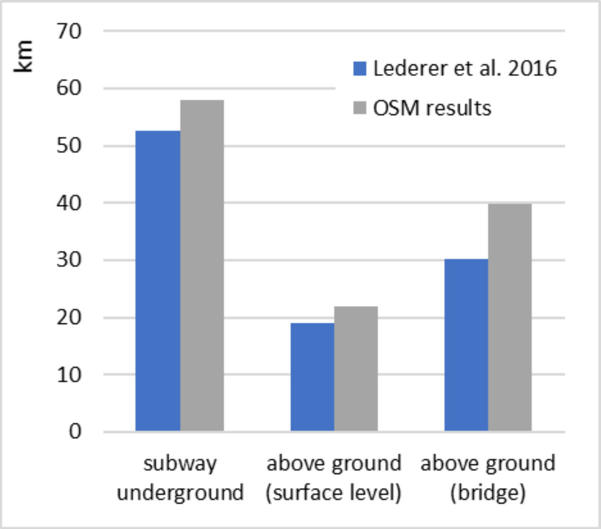

Figure SI_7. Comparison of subway length data from OSM versus data from Lederer et al. [15]

3. Material intensities of buildings and infrastructures

3.1 Definition of volumes used to estimate material intensities

Building research usually defines the gross-building-volume for a building as depicted in SI_8a. Often, buildings research also uses floor area, measured in square meters of living space (excluding most walls, sometimes also communal areas, etc.). Material intensities for buildings are then developed depending on the definition of a building. The modelled building volumes as derived from the satellite-data and machine-learning modelling, however requires and uses an above-ground definition of a building, as shown in Figure SI_8b. The derived modelled-building-volume is used for the further estimations presented in this article. All material intensities for Germany and Austria, which were originally derived from a buildings definition shown in a), are re-calculated to use the building volumes definition shown in b).

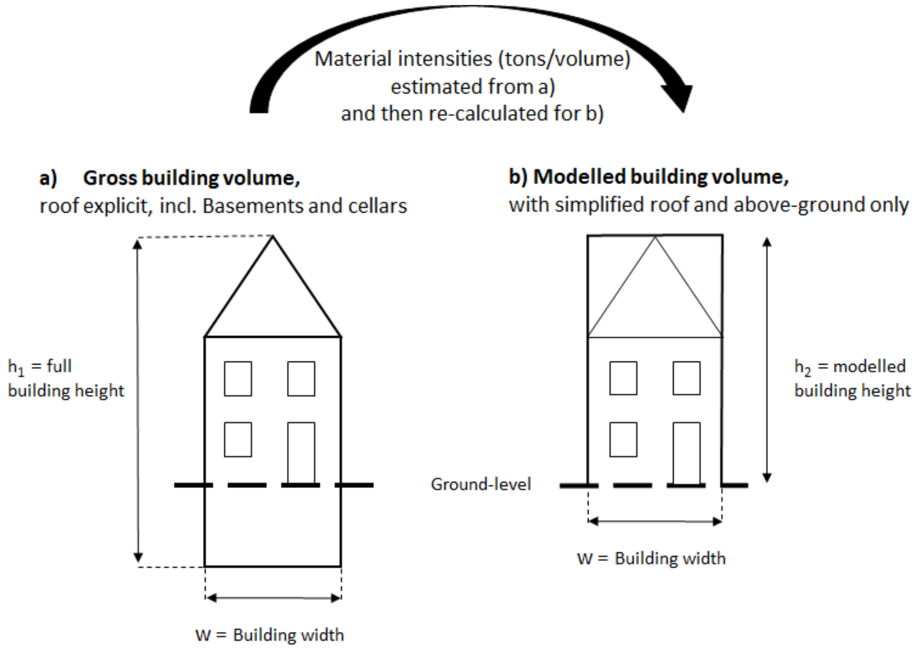

Figure SI_8. The relation between the common building definitions of gross building volume, and the definition of above-ground modelled building volume as used for this study.

3.2 Material intensity factors

The dataset of material intensities (MI) was compiled and re-estimated using information from different sources. If possible, country-specific values were used. In the following, we shortly summarize the rationale and sources used for each stock-type and if applicable, the specifics for Austria and Germany specific MIs. For buildings, in this study, we use modelled-above ground building volumes as described in Figure SI_1, which makes it necessary to re-calculate material intensities from existing studies for the sake of consistency. For Germany, material intensities (tons/m^3) for all residential building types were re-estimated from literature sources[16,17]. For lightweight buildings, data from Switzerland[18,19] were used. For commercial/industrial buildings, re-estimation into modelled-above ground building volumes was not feasible due to data limitations and the building definition of Figure SI_1a was used. Sources used for constructing the MI-dataset for Germany were the IOER-Database[16] and Ortlepp et al. [20]. For Austria, material intensities for a sample of buildings are available for Vienna from Lederer et al.[21]. This Vienna-specific information on single-family residential buildings, multi-family residential buildings and commercial/industrial buildings was then weighted using national statistics about Austrian building stock. For lightweight buildings and high-rise multi-family residential buildings, no

Austria-specific data was available and material intensities from Germany[16] and from Switzerland[18] were used.

For road infrastructures, material intensities applicable to both Austria and Germany were developed, as we assumed that there would be no fundamental difference between roads in these two neighboring countries. We collected all available studies quantifying the asphalt top layers and aggregate base-courses required to build 'typical' roads and averaged across these studies, as material intensities for roads are a notoriously underdetermined issue[5,9,10,22,23]. When material intensities are only available per km of road length[5,10], a re-calculation into intensities per m^2 was necessary. For this recalculation, we used average widths per road type (Table SI_4). For airport runways, taxiways and airplane parking areas we assumed the same material intensity as for motorways.

For bridges, material intensities were sourced from Gassner et al.[24], who gives information about the material intensity of Viennese bridges without the road surface. The mass of the asphalt layer of a road on top of a bridge was sourced from the available material intensities of roads as described above. For tunnels, material intensities for Germany are derived from Steger et al.[9], which are available in tons/km. This source was used for road- and railway-tunnels in both Germany and Austria. For this study, we re-calculated tons/km into tons/m^2 of tunnel and used country-specific information from OSM on the length and width of all 32 road types in tunnels to derive a weighted average tunnel-width.

For railways, material intensities in t/km single-track are available for Germany[9]. The types and amounts of sleepers (concrete, wood and steel) and the share of slab tracks was varied between Austria and Germany using additional sources[25,26]. Data on railway bridges was taken from Steger et al.[9].

For subways, material intensities are available for Vienna[15]. Using the split of subway tracks running underground (OSM-tag: tunnel), on ground level (neither bridge nor tunnel OSM-tag) and elevated on stilts (OSM-tag: bridge), material intensities were refined for these three different types of subways. For trams, material intensities are available for Vienna[27] and the other track types (<5% of total length including "light rails", "narrow gauge" and "preserved") were

calculated with the same material intensity for trams. For all types of tracks, a re-calculation of data per km length into shares per m^2 surface was made, multiplying by the average width of tracks as used in the mapping.

Material intensity values used in the calculation are reported in the Supplementary Data Spreadsheet.

5. Comparison of mapped building volumes with statistical data

Using statistical information about buildings in Germany and Austria, an estimation of building volumes was calculated and compared to the results of this analysis. As data structures in German and Austrian statistics are slightly different, a process was designed that allowed a similar approximation of building volumes in both countries.

5.1 Building volumes in Germany

Figure SI_9 gives an overview of the methodology used to calculate building volumes based on statistical data. Conversion factors used to extrapolate gross floor area from usable floor area are reported in Table SI_6.

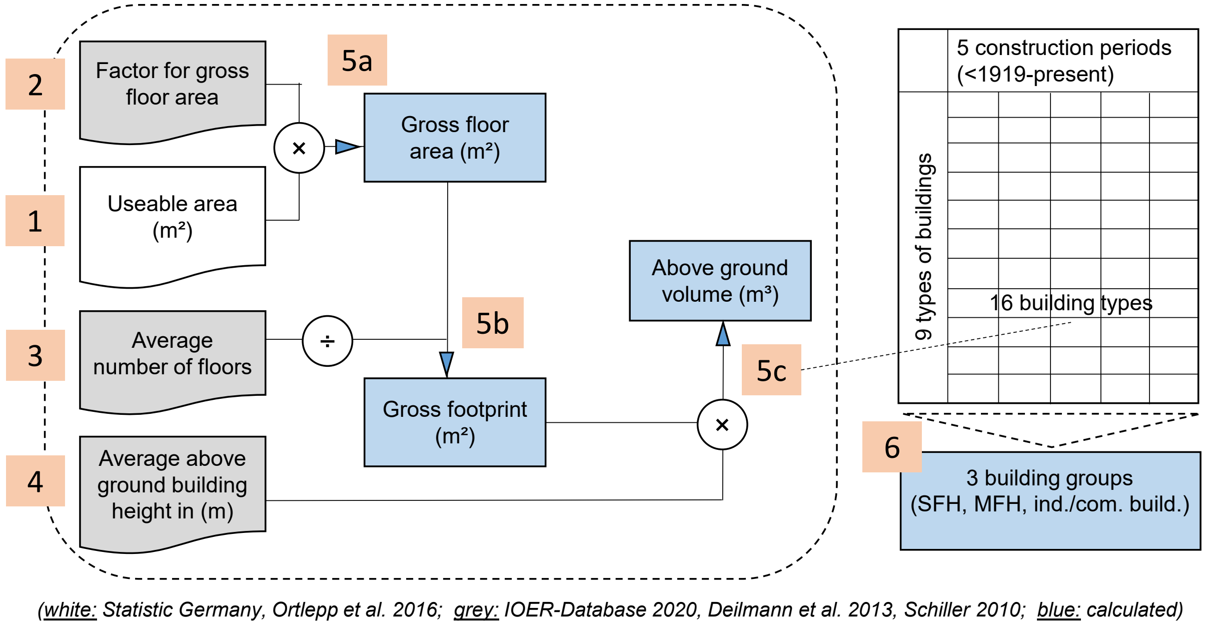

Figure SI_9: Methodology for calculating building volumes in Germany from statistical data following six steps (see boxes)

In the following text, bold numbers refer to the numbers of pink boxes in Figure SI_9. **(1)** Sixteen building types were examined, as displayed in Table SI_6. Usable floor areas (according to the literature[28,29] and from Destatis[30]) for different buildings were grouped into building types and construction periods[31] for all residential buildings. For non-residential buildings, only data for new construction and demolition[20] was available. **(2)** The gross floor area was calculated on basis of shares (ratios) between useable area (e. g. for living, office, factory, store etc.) and gross floor area[16], see Table SI_6. **(3)** The average number of floors (including attics/roofs and basements) was taken from the literature[16,32,33]. **(4)** The average building height for residential buildings was taken from[16,32]. Step **(5)** is explained below in more detail. **(6)** At the end of the volume calculations, the volumes of the 16 building types are aggregated to three building groups: SFH, MFH and industrial/commercial buildings. Table SI_6, column 1 shows the assignments/allocations.

Table SI_6. Factors for conversion of usable area into gross floor area [30], average number of floors [16,32,33], and above-ground building height per building type

**Statistical building types (Germany)**	**Share of useable area in gross floor area**	**Number of floors**	**Above ground building height (incl. roof as box)**
SFH until 1948	0.74	2.5	8.74
SFH 1949-78	0.75	2.5	8.09
SFH 1979-90	0.76	2.5	8.29
SFH from 1991	0.80	2.5	9.17
MFH until 1918	0.71	5.0	15.28
MFH 1919-48	0.74	5.1	15.72
MFH 1949-78	0.77	5.6	16.10
MFH 1979-90	0.78	5.9	17.26
MFH from 1991	0.82	4.7	14.84
Institutional buildings	0.64	3.6	9.39
office/administrative buildings	0.66	4.4	10.08
agricultural commercial buildings	0.89	1.0	5.80
factory/workshop buildings	0.75	1.8	6.59
trade/storage buildings	0.86	1.6	6.00
hotels/restaurants	0.65	3.9	8.06
other non-residential buildings	0.65	2.8	8.41

In step (5), using this information derived in the previous steps was used to calculate ground building volumes for all 16 building types and in the end clustered into three building types (**5a,b,c**):

a) Gross floor area per building type

$$gfa_{bt} = \sum_{k=1}^{n} ua_{bt,k} \times sua_{bt,k}$$

gfa_{bt} gross floor area of building type [m²]
ua_{bt} useable area of building type [m²]
sua_{bt} Share of useable area in gross floor area of building type [-]

b) Gross footprint per building type

$$gf_{bt} = \sum_{k=1}^{n} gfa_{bt,k} \div nf_{bt,k}$$

gf_{bt} gross footprint of building type [m²]
gfa_{bt} gross floor area of building type [m²]
nf_{bt} number of floors of building type [-]

c) above ground volume per building type

$$agv_{bt} = \sum_{k=1}^{n} gf_{bt,k} \times agbh_{bt,k}$$

agv_{bt} above ground volume of building type [m³]
gf_{bt} gross footprint of building type [m²]
$agbh_{bt}$ above ground building height of building type [m]

As displayed in Figure SI_10, the comparison of the building volumes for Germany shows that the mapping data is on average 41% larger than that estimated by us using statistical data (statistical estimates = 100%). The mapping values are 112% larger for single family houses (without garages/lightweight buildings), 75% larger for multi-family houses and 14% lower for non-residential buildings.

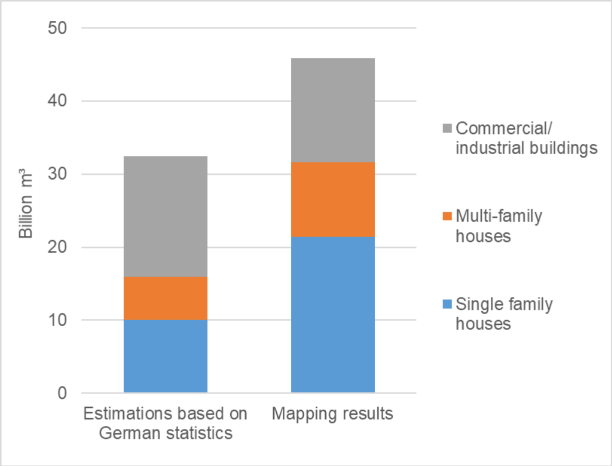

Figure SI_10. Mapped building volumes in Germany are 41% higher than results derived from statistics

5.2 Building volumes in Austria

Figure SI_11 gives an overview of the methodology used to calculate building volumes based on statistical data. Conversion factors used to extrapolate gross floor area from usable floor area are reported in Table SI_7.

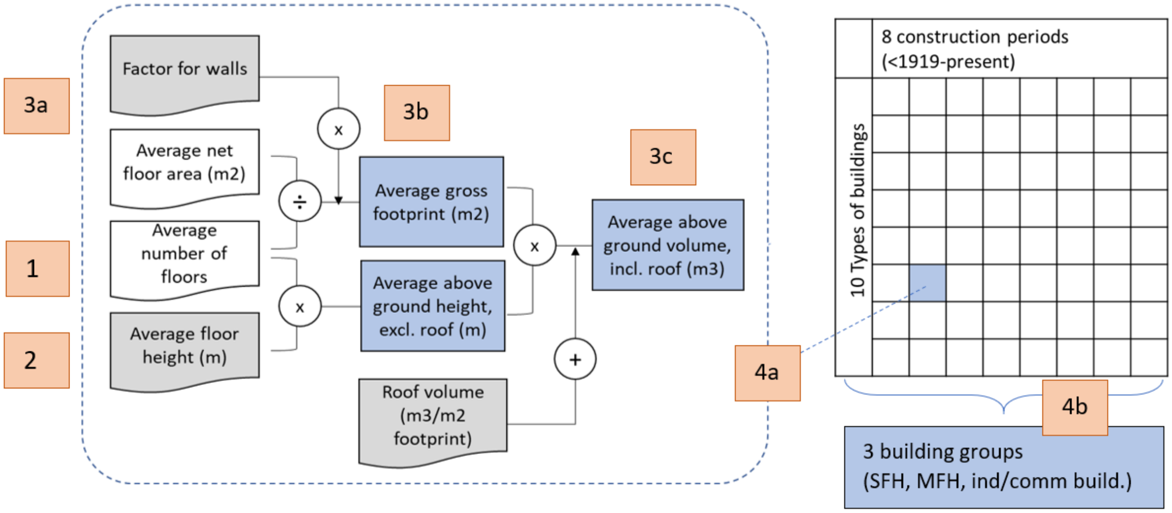

Figure SI_11. Methodology for calculating building volumes in Austria from statistical data following four steps (orange boxes). White boxes represent data from[34], grey boxes from [21], blue boxes are results from calculations

In the following text, bold numbers refer to the numbers of pink boxes in Figure SI_11: **(1)** The average number of floors for every building type in every building period and every building

size-class was calculated, weighted by the number of buildings existing in this group[34]. Buildings with >11 floors were assumed to have 15 floors on average. **(2)** The average height per building type for each size-class and building period was calculated by multiplying the number of floors with the average height per floor[21], see Table SI_7. Multiplying the average number of floors and the average height per floor delivered the average above ground height per building excluding the roof. **(3a)** The net footprint (excluding walls) of the building was calculated by using the average usable floor space divided by the number of floors for every size class and building period. Buildings with usable floor area of >1,000 m^2 were assumed to have a usable floor area of 1,500 m^2. **(3b)** A factor for converting the usable floor area into gross footprint area[21] was used to derive gross footprints for all building types. **(3c)** Volumes of buildings without roofs (cuboids) were calculated by multiplying gross footprint and average height of each type of building. The volume of the roof was calculated by using a factor[21] of m^3 roof volume per m^2 gross footprint for each building type and period (see Table SI_9). Both volumes were added to arrive at total building volumes. **(4a)** Building volumes for each building type and period were multiplied by the number of buildings registered in Austria in each size-class and building period[34]. **(4b)** The results were then clustered in building type groups.

Table SI_7. Average heights per floor[21]

**Buildings**		**Average height per floor**				
**Group**	**Building types**	**< 1919**	**1919-1945**	**1946-1976**	**1977-1996**	**> 1996**
Single-family houses	Residential buildings with 1 unit	3.01	2.69	2.76	2.78	2.86
	Residential buildings with 2 units	3.01	3.01	3.01	3.01	3.01
Multi-family houses	Residential buildings with =>3 units	3.46	3.46	3.46	3.46	3.46
	Buildings for communities	3.59	3.59	3.59	3.59	3.59
Industrial & commercial buildings	Hotels and similar buildings	3.95	3.95	3.95	3.95	3.95
	Office buildings	3.95	3.95	3.95	3.95	3.95
	Commercial buildings	3.95	3.95	3.95	3.95	3.95
	Traffic administration/news buildings	3.85	3.85	3.85	3.85	3.85
	Industrial and storage buildings	3.85	3.85	3.85	3.85	3.85
	Buildings for cultural, recreational, education and health care purposes	3.95	3.95	3.95	3.95	3.95

Table SI_8. Factors for conversion of usable floor area into gross footprint area[21]

Buildings		Share of usable floor area in gross floor area				
**Group**	**Building types**	**< 1919**	**1919-1945**	**1946-1976**	**1977-1996**	**> 1996**
Single-family houses	Residential buildings with 1 unit	0.78	0.78	0.79	0.79	0.78
	Residential buildings with 2 units	0.78	0.78	0.79	0.79	0.78
Multi-family houses	Residential buildings with =>3 units	0.78	0.79	0.81	0.77	0.76
	Buildings for communities	0.75	0.82	0.84	0.86	0.84
Industrial & commercial buildings	Hotels and similar buildings	0.75	0.77	0.92	0.90	0.95
	Office buildings	0.75	0.77	0.92	0.90	0.95
	Commercial buildings	0.75	0.77	0.92	0.90	0.95
	Traffic administration/news buildings	0.62	0.78	0.87	0.81	0.72
	Industrial and storage buildings	0.62	0.78	0.87	0.81	0.72
	Buildings for cultural, recreational, education and health care purposes	0.75	0.77	0.92	0.90	0.95

Table SI_9. Additional volume of roofs in m^3/m^2 gross footprint[21]

Buildings		m3 of roof volume per m2 of gross footprint				
**Group**	**Building types**	**< 1919**	**1919-1945**	**1946-1976**	**1977-1996**	**> 1996**
Single-family houses	Residential buildings with 1 unit	1.74	1.87	1.82	0.80	1.13
	Residential buildings with 2 units	1.74	1.87	1.82	0.80	1.13
Multi-family houses	Residential buildings with =>3 units	2.34	2.91	1.44	1.61	1.42
	Buildings for communities	3.57	1.53	1.35	0.80	0.84
Industrial & commercial buildings	Hotels and similar buildings	1.16	2.74	0.20	0.17	0.16
	Office buildings	1.16	2.74	0.20	0.17	0.16
	Commercial buildings	1.16	2.74	0.20	0.17	0.16
	Traffic administration/news buildings	1.69	0.31	0.43	0.13	0.12
	Industrial and storage buildings	1.69	0.31	0.43	0.13	0.12
	Buildings for cultural, recreational, education and health care purposes	1.16	2.74	0.20	0.17	0.16

Figure SI_12 provides a comparison of building volumes per building type in Austria with an estimate derived from combining available statistical data for buildings in Austria. Results from our mapping are 74% higher than the results derived from statistical data. Mapped volumes of single-family houses are 70% larger (statistical estimates = 100%), volumes of multi-family houses are 57% larger and commercial and industrial buildings are 99% larger than volumes derived from statistics. Statistical data are from the year 2011 and do not capture some kinds of buildings such as agriculturally used buildings or buildings which are not in use any more (hibernating stock). The calculation of roof volumes in statistical results represent real roof volumes while in the mapping, roof volumes were added "as a box" as illustrated in Fig SI_8, this is another factor contributing to the difference. Further reasons for this difference could be

underestimations in the factors for gross floor area, roof volume and average floor heights or in the assumptions about number of floors or estimated floor space used.

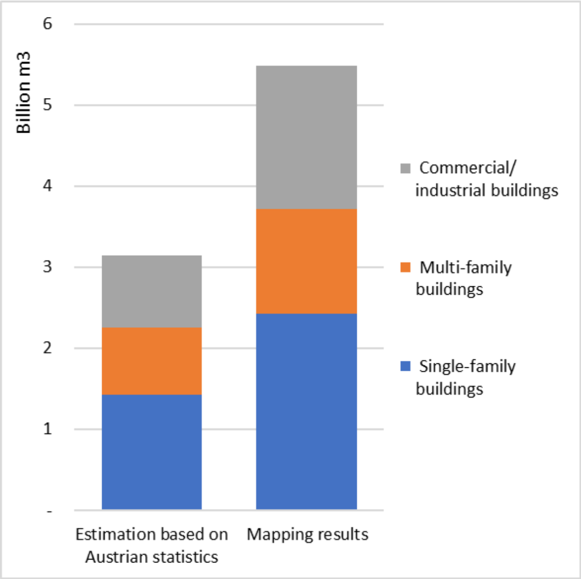

Figure SI_12. Mapped building volumes in Austria are 74% higher than results derived from statistical data[34]

6. Additional results: Detailed material stock data for Germany and Austria

Additional details on the results on material stocks in Austria and Germany (e.g. more detailed breakdowns of material types than available in the main text) are reported in the Supplementary Data Spreadsheet (SDS).

Figure SI_13 shows the estimated distribution of total material stocks in 2D at a spatial resolution of 100 m for the two countries of our study area, Austria and Germany. It complements Figure 2 in the article that shows a 3D presentation.

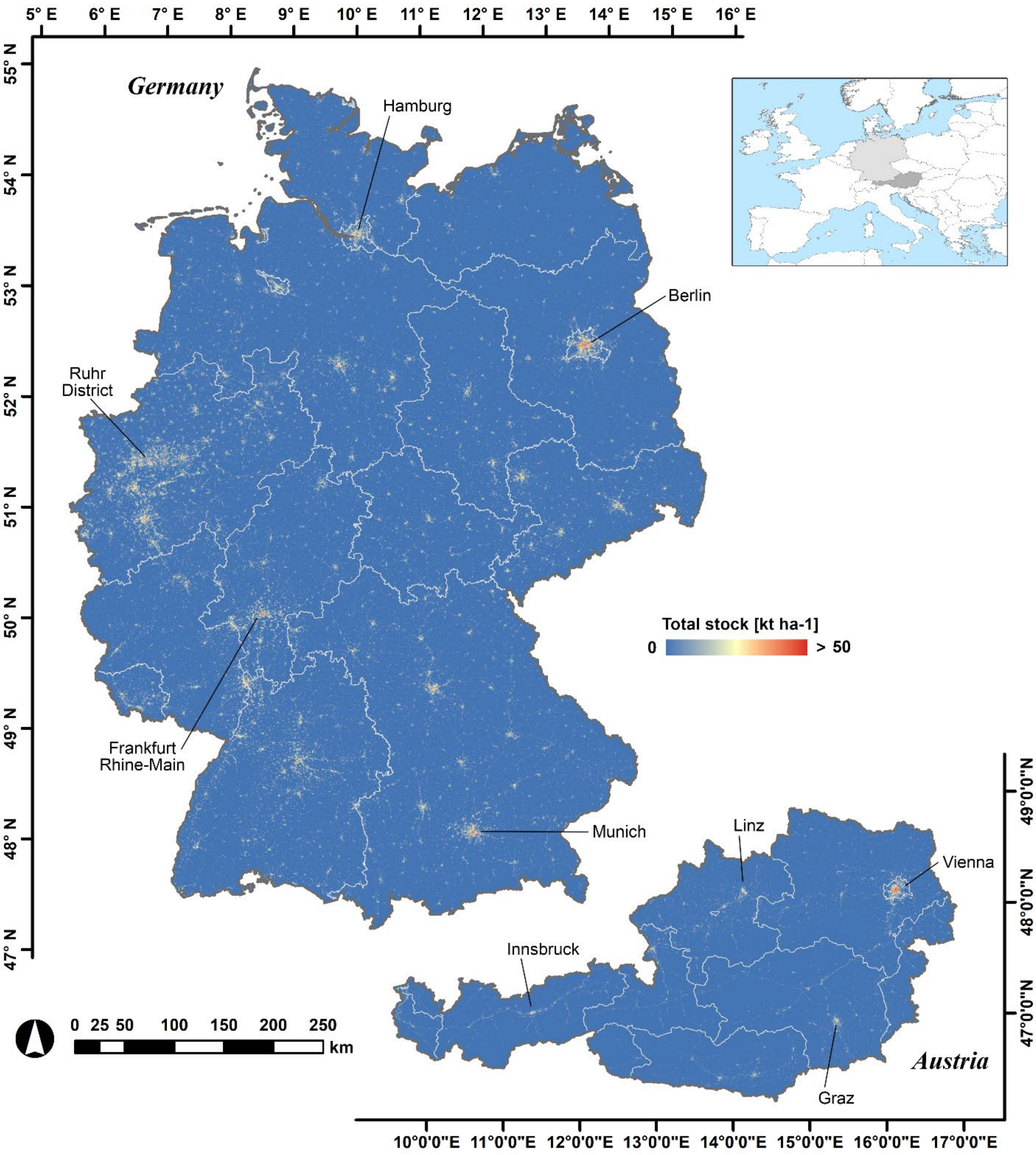

Figure SI_13. Two-dimensional maps of total material stocks in buildings and infrastructures in Germany and Austria (2018; 100m resolution), measured as kt/ha (1 kt = 1,000 metric tons; 1 ha = 10^4 m^2 = 0.01 km^2).

References

(1) Schug, F.; Frantz, D.; Okujeni, A.; van der Linden, S.; Hostert, P. Mapping Urban-Rural Gradients of Settlements and Vegetation at National Scale Using Sentinel-2 Spectral-Temporal Metrics and Regression-Based Unmixing with Synthetic Training Data. *Remote Sensing of Environment* **2020**, *246*, 111810. https://doi.org/10.1016/j.rse.2020.111810.

(2) Frantz, D.; Schug, F.; Okujeni, A.; Navacchi, C.; Wagner, W.; van der Linden, S.; Hostert, P. National-Scale Mapping of Building Height Using Sentinel-1 and Sentinel-2 Time Series. *Remote Sensing of Environment* **2021**, *252*, 112128. https://doi.org/10.1016/j.rse.2020.112128.

(3) Schug, F.; Frantz, D.; van der Linden, S.; Hostert, P. Large Area Gridded Population Mapping Based on Building Density, Height and Type from Earth Observation Data Using Census Disaggregation and Bottom-up Estimates. *PLoS One in review*.

(4) Leibniz-Institut für ökologische Raumentwicklung. *Monitor Der Siedlungs- Und Freiraumentwicklung (IÖR-Monitor): Pufferung von Linienobjekten*; https://www.ioer-monitor.de/methodik/#c244 (accessed 08.04.2020): Leipzig, 2020.

(5) Wiedenhofer, D.; Steinberger, J. K.; Eisenmenger, N.; Haas, W. Maintenance and Expansion: Modeling Material Stocks and Flows for Residential Buildings and Transportation Networks in the EU25: Stocks and Flows in the EU25. *Journal of Industrial Ecology* **2015**, *19* (4), 538–551. https://doi.org/10.1111/jiec.12216.

(6) Bundesamt für Kartographie und Geodäsie. *Dokumentation Digitales Basis-Landschaftsmodell Basis-DLM*; https://www.bkg.bund.de/SharedDocs/Produktinformationen/BKG/DE/P-2019/190114_ATKISDLM.html, 2020.

(7) Meijer, J. R.; Huijbregts, M. A. J.; Schotten, K. C. G. J.; Schipper, A. M. Global Patterns of Current and Future Road Infrastructure. *Environ. Res. Lett.* **2018**, *13* (6), 064006. https://doi.org/10.1088/1748-9326/aabd42.

(8) Statistisches Bundesamt Destatis. Verkehrsinfrastruktur in Deutschland, 2017.

(9) Steger, S.; Fekkak, M.; Bringezu, D. S. Materialbestand und Materialflüsse in Infrastrukturen. Meilensteinbericht des Arbeitspakets 2.3 des Projekts "Materialeffizienz und Ressourcenschonung" (MaRess). **2011**, 268.

(10) Knappe, F.; Reinhardt, J.; Bergmann, T.; Mottschall, M. Substitution von Primärrohstoffen im Straßen- und Wegebau durch mineralische Abfälle und Bodenaushub; Stoffströme und Potenziale unter Berücksichtigung von Neu-, Aus- und Rückbau sowie der Instandsetzung. **2015**, 95.

(11) BMVIT. *Statistik Straße und Verkehr*; Wien, 2020; p 45.

(12) Deutsche Bahn AG. Geschäftsbericht, 2020.

(13) ÖBB. *Zahlen Daten Fakten*; 2020.

(14) Statistik Austria. Schieneninfrastruktur in Österreich, 2019.

(15) Lederer, J.; Kleemann, F.; Ossberger, M.; Rechberger, H.; Fellner, J. Prospecting and Exploring Anthropogenic Resource Deposits: The Case Study of Vienna's Subway Network: Prospecting and Exploring Anthropogenic Resource Deposits. *Journal of Industrial Ecology* **2016**, *20* (6), 1320–1333. https://doi.org/10.1111/jiec.12395.

(16) IOER-Database. Information Portal for Structural Data, 2020.

(17) Ortlepp, R.; Gruhler, K.; Schiller, G. Materials in Germany's Domestic Building Stock: Calculation Model and Uncertainties. *Building Research & Information* **2018**, *46* (2), 164–178. https://doi.org/10.1080/09613218.2016.1264121.

(18) Mouron, P. *Garage Construction, Wood, Non-Insulated, Fire- Protected, CH, Undefined System Model*; Ecoinvent Database v3.4: https://www.ecoinvent.org, 2016.

(19) Lanau, M.; Liu, G. Developing an Urban Resource Cadaster for Circular Economy: A Case of Odense, Denmark. *Environ. Sci. Technol.* **2020**, *54* (7), 4675–4685. https://doi.org/10.1021/acs.est.9b07749.

(20) Ortlepp, R.; Gruhler, K.; Schiller, G. Material Stocks in Germany's Non-Domestic Buildings: A New Quantification Method. *Building Research & Information* **2016**, *44* (8), 840–862. https://doi.org/10.1080/09613218.2016.1112096.
(21) Lederer, J.; Fellner, J.; Gassner, A.; Gruhler, K.; Schiller, G. Determining the Material Intensities of Buildings Selected by Random Sampling: A Case Study of Vienna. *Journal of Industrial Ecology* **2021**, *in press*.
(22) Virág, D. *Das Verkehrssystem im Stock-Flow-Service-Nexus. Analyse der Materialbestände und -flüsse für verschiedene Formen von Mobilität in Wien*; Social Ecology Working Paper No. 179: Vienna, 2019.
(23) Virág, D.; Wiedenhofer, D.; Haas, W.; Haberl, H.; Kalt, G.; Krausmann, F. The Stock-Flow-Service Nexus of Personal Mobility in an Urban Context: Vienna, Austria. *Environment and Development in review*.
(24) Gassner, A.; Lederer, J.; Fellner, J. Material Stock Development of the Transport Sector in the City of Vienna. *Journal of Industrial Ecology* **2020**. https://doi.org/10.1111/jiec.13024.
(25) Haban, F. X. Theoretische und experimentelle Untersuchungen an Spannbetonschwellen. 2016.
(26) Holzforschung Austria. Imprägnierung von Buchenholzschwellen Mit Potentiellen Kreosot-Alternativen, 2018.
(27) Gassner, A.; Lederer, J.; Kanitschar, G.; Ossberger, M.; Fellner, J. Extended Ecological Footprint for Different Modes of Urban Public Transport: The Case of Vienna, Austria. *Land Use Policy* **2018**, *72*, 85–99. https://doi.org/10.1016/j.landusepol.2017.12.012.
(28) ISO 9836 2017-09. *Performance Standards in Building - Definition and Calculation of Area and Space Indicators*.
(29) Schiller, G.; Miatto, A.; Gruhler, K.; Ortlepp, R.; Deilmann, C.; Tanikawa, H. Transferability of Material Composition Indicators for Residential Buildings: A Conceptual Approach Based on a German-Japanese Comparison. *Journal of Industrial Ecology* **2019**, *23*, 796–807.
(30) Destatis. *Bestand an Wohnungen - Fachserie 5 Reihe 3*; 2018.
(31) Destatis. *Bauen Und Wohnen. Mikrozensus – Zusatzerhebung 2014. Fachserie 5 Heft 1*; Statistisches Bundesamt: Wiesbaden, 2016; p 457.
(32) Deilmann, C.; Behnisch, M.; Dirlich, S.; Gruhler, K.; Hagemann, U.; Petereit, R.; Kunz, C.; Petereit, K. *Systematische Datenanalyse Im Bereich Der Nichtwohngebäude - Erfassung Und Quantifizie-Rung von Energieeinspar- Und CO2-Minderungspotenzialen*; BMVBS: Berlin, 2013; p 120.
(33) Schiller, G. Kostenbewertung Der Anpassung Zentraler Abwasserentsorgungssysteme Bei Bevölkerungs-Rückgang. *IÖR-Schriften* **2010**, *51*, 254. http://dx.doi.org/10.1080/09613218.2016.1112096.
(34) Statistik Austria. *Registerzählung 2011 - Gebäude Und Wohnungszählung*; STATcube - Statistische Datenbank von Statistik Austria; 2011.
